# Deployment of Mobile EEG Technology in an Art Museum Setting: Evaluation of Signal Quality and Usability

**DOI:** 10.3389/fnhum.2017.00527

**Published:** 2017-11-10

**Authors:** Jesus G. Cruz-Garza, Justin A. Brantley, Sho Nakagome, Kimberly Kontson, Murad Megjhani, Dario Robleto, Jose L. Contreras-Vidal

**Affiliations:** ^1^Laboratory for Non-Invasive Brain Machine Interfaces, Department of Electrical and Computer Engineering, University of Houston, Houston, TX, United States; ^2^Office of Science and Engineering Laboratories, Division of Biomedical Physics, Center for Devices and Radiological Health, U.S. Food and Drug Administration, Silver Spring, MD, United States; ^3^Department of Neurology, Columbia University, New York, NY, United States; ^4^Cullen College of Engineering, Houston, TX, United States

**Keywords:** MoBI, aesthetics, EEG, dry-electrodes, signal quality, museum, real-world recording

## Abstract

Electroencephalography (EEG) has emerged as a powerful tool for quantitatively studying the brain that enables natural and mobile experiments. Recent advances in EEG have allowed for the use of dry electrodes that do not require a conductive medium between the recording electrode and the scalp. The overall goal of this research was to gain an understanding of the overall usability and signal quality of dry EEG headsets compared to traditional gel-based systems in an unconstrained environment. EEG was used to collect Mobile Brain-body Imaging (MoBI) data from 432 people as they experienced an art exhibit in a public museum. The subjects were instrumented with either one of four dry electrode EEG systems or a conventional gel electrode EEG system. Each of the systems was evaluated based on the signal quality and usability in a real-world setting. First, we describe the various artifacts that were characteristic of each of the systems. Second, we report on each system's usability and their limitations in a mobile setting. Third, to evaluate signal quality for task discrimination and characterization, we employed a data driven clustering approach on the data from 134 of the 432 subjects (those with reliable location tracking information and usable EEG data) to evaluate the power spectral density (PSD) content of the EEG recordings. The experiment consisted of a baseline condition in which the subjects sat quietly facing a white wall for 1 min. Subsequently, the participants were encouraged to explore the exhibit for as long as they wished (piece-viewing). No constraints were placed upon the individual in relation to action, time, or navigation of the exhibit. In this freely-behaving approach, the EEG systems varied in their capacity to record characteristic modulations in the EEG data, with the gel-based system more clearly capturing stereotypical alpha and beta-band modulations.

## Introduction

Technological advances in Mobile Brain-body Imaging (MoBI) technology now allow the study of natural cognition and action in real-world complex environments. In this type of deployment, MoBI systems require synchronous recordings of brain activity, environment capture technology (such as cameras that make recordings context-aware), and recording of internal or external events influencing cognition and action (Gramann et al., 2014b). MoBI technology is expected to overcome technical constraints arising from limitations of traditional brain/body imaging modalities that restrict subjects to limited or no movement during cognitive or motor tasks. This restriction may contradict the very goal of such studies, which seek to elucidate the underlying neural activity “in action and in context” involved in natural human cognition or movement. Furthermore, cognitive processes are often based on the surrounding environment and our own physical body (Wilson, 2002). This has implications in both perceptual and motor processing, and may affect, for example, the way we perceive a viewed art piece or prepare to execute a goal-oriented movement. Thus, it is imperative to deploy both hardware and software that allow for the simultaneous, reliable, and user-friendly recording of brain activity and body movements during mobile applications.

A primary challenge in implementing MoBI protocols is the development of wearable neurotechnology that allows for high-quality recordings of brain responses and other physiological and environmental signals associated with human experiences and behaviors in natural complex settings. Over the last several years, the development of “mobile friendly” neuroimaging modalities, such as EEG and functional near-infrared spectroscopy (fNIRS), has advanced our understanding of neurocognitive processes during perceptual (Makeig et al., 2009; Gramann et al., 2014a; Jungnickel and Gramann, 2016) and motor tasks, such as locomotion on a treadmill (Gwin et al., 2011; Presacco et al., 2011, 2012; Wagner et al., 2014; Bulea et al., 2015), transition between movement states (i.e., movement intent) (Bulea et al., 2014), expressive movement (Cruz-Garza et al., 2014), and multi-terrain over-ground locomotion (Brantley et al., 2016). Moreover, these technologies provide a sensitive and reliable index to assess brain activity involved in skill acquisition, task performance, and during the development of expertise in complex tasks (Ayaz et al., 2013). Recently, the use of these systems has uncovered brain dynamics during aesthetic experiences in a real-world environment (Kontson et al., 2015; Kovacevic et al., 2015). New applications of these MoBI systems are being discovered on a regular basis. However, each modality brings with it technological challenges that potentially threaten the integrity of the data collected in these real-world settings. For example, locomotion and external environmental factors may adversely affect EEG data recorded under a MoBI protocol. Additionally, the usability and fit of various headsets may influence the quality of the data collected under these MoBI conditions.

Until recently, EEG recordings were limited to systems that require a conductive medium to be inserted between the recording electrode and the individual's scalp (e.g., saline, gels, etc.). These systems are somewhat cumbersome and laborious in their set-up, and leave the subject with gelatinous residue in their hair (Ferree et al., 2001). Continuous use of gel on the skin might also result in allergic reactions or infections (Griss et al., 2002). Recent advancements in electrode technology have led to the development of dry electrode systems, which may allow for (1) reduced set-up time, (2) greater subject mobility, and (3) signal quality equivalent to that of the gel-based systems (Zander et al., 2011; Guger et al., 2012; Liao et al., 2012; Mihajlović et al., 2015; Oliveira et al., 2016). EEG is susceptible to signal contamination from non-physiological and physiological sources, including power-line interference, electrode pops, ocular motions (e.g., eye blink, saccades, and fixations), muscle activation, and cardiac activities (Mahajan and Morshed, 2015). Dry EEG systems may be more susceptible to these artifactual phenomena due to an overall increased interface impedance and potential loss of a stable contact between the scalp and the recording electrode (Guger et al., 2012; Laszlo et al., 2014).

Previous studies have evaluated dry and gel based systems for signal acquisition and quality (Chi et al., 2012; Oliveira et al., 2016; Wang et al., 2016), comfort, head size adaptability and stability of electrode-scalp electrical connection during cognitive tasks (Hairston et al., 2014). Chi et al. used sensor correlation between the wet and the dry signals, power spectral density (PSD), and signal to noise ratio (SNR) for the comparison among the wet and dry electrodes and concluded that although the dry systems suffer from signal degradation, they were feasible for basic brain-computer interface (BCI) applications (Chi et al., 2012). Oliveira et al. also proposed a metric to benchmark the suitability of upcoming EEG technologies which were based on a comparison of the signal-to-noise ratio, EEG amplitude variance, and event related potentials (ERPs) between wet and dry EEG systems (Oliveira et al., 2016). Contrary to what Chi et al. reported (Chi et al., 2012), Oliveira et al. concluded that the dry EEG systems may need substantial improvement in order to match the quality of wet systems (Oliveira et al., 2016). Another recent study from Wang et al. compared power spectra and time-frequency maps (spectrograms) between the two systems to evaluate the feasibility of novel semi-dry electrodes (Wang et al., 2016). Although these novel system designs show promise in a controlled laboratory setting, few studies have shown the feasibility of individuals using these systems in a dynamic, real-world environment (Gargiulo et al., 2008; Liao et al., 2012; Lin et al., 2013; Yeung et al., 2015).

In this study, EEG recordings were collected from 432 individuals as they experienced an art exhibit in a museum. The subjects were instrumented with one of four dry electrode EEG systems, or a conventional gel electrode EEG system. Each of the systems was evaluated based on the signal quality and usability in a real-world setting. A description of each system is provided, with details on the electrode montage, electrode type, and technical specifications. First, we report on the typical artifacts identified with each system type, and the probable cause(s) of each artifact. Second, we evaluated the spectral content of EEG recordings during art viewing and a control condition across the dry electrode systems and the conventional gel electrode system using a data driven clustering approach. Third, we examined patterns in spectral content across the EEG headsets to compare between task conditions.

The overall goals of this research are to gain an understanding of the overall usability and signal quality of dry EEG headsets in an unconstrained environment. The usability and signal quality of the systems are described in terms of the expected artifacts to find in the experimental setup, the limitations of the systems, and in the analysis of PSD patterns taken from the different headsets. Furthermore, this study may provide a guideline for future non-laboratory studies using mobile brain imaging technologies.

## Materials and methods

### Participants

The experimental protocol and anonymous informed consent were approved by the University of Houston's Institutional Review Board (IRB) to minimize the disruption of the museum environment and protect the privacy of the participants. Adults volunteers provided anonymous informed verbal consent prior to participating in the experiment. Children between the ages of 6 and 18 years old provided anonymous assent to participate in the experiment, whereas their parent/guardian, provided anonymous informed verbal consent to have their child participate. Written informed consent/parental consent was not obtained because the IRB Committee waived the requirement to obtain a signed consent form for the participants as the only record linking the subject and the research would be the consent document. Thus, the principal risk would be potential harm resulting from a breach of confidentiality.

The experiments were conducted weekly at The Menil Collection (Houston, TX) over the course of a 4-month period. Visitors to the museum, ages 6 years and older, were invited to view Dario Robleto's installation at The Menil Collection, *The Boundary of Life is Quietly Crossed*, while wearing a MoBI system. All participants provided verbal consent to participate in the study and were asked to refrain from touching the headset. A total of 432 (134 with reliable location tracking information and usable EEG data) museum visitors participated as on-site volunteers for this experiment. The demographic distribution of the volunteers was comparable to the demographics in the population of the Houston Metropolitan area, Texas (Supplementary Figure 1).

### Experimental set-up

**Figure 1:**
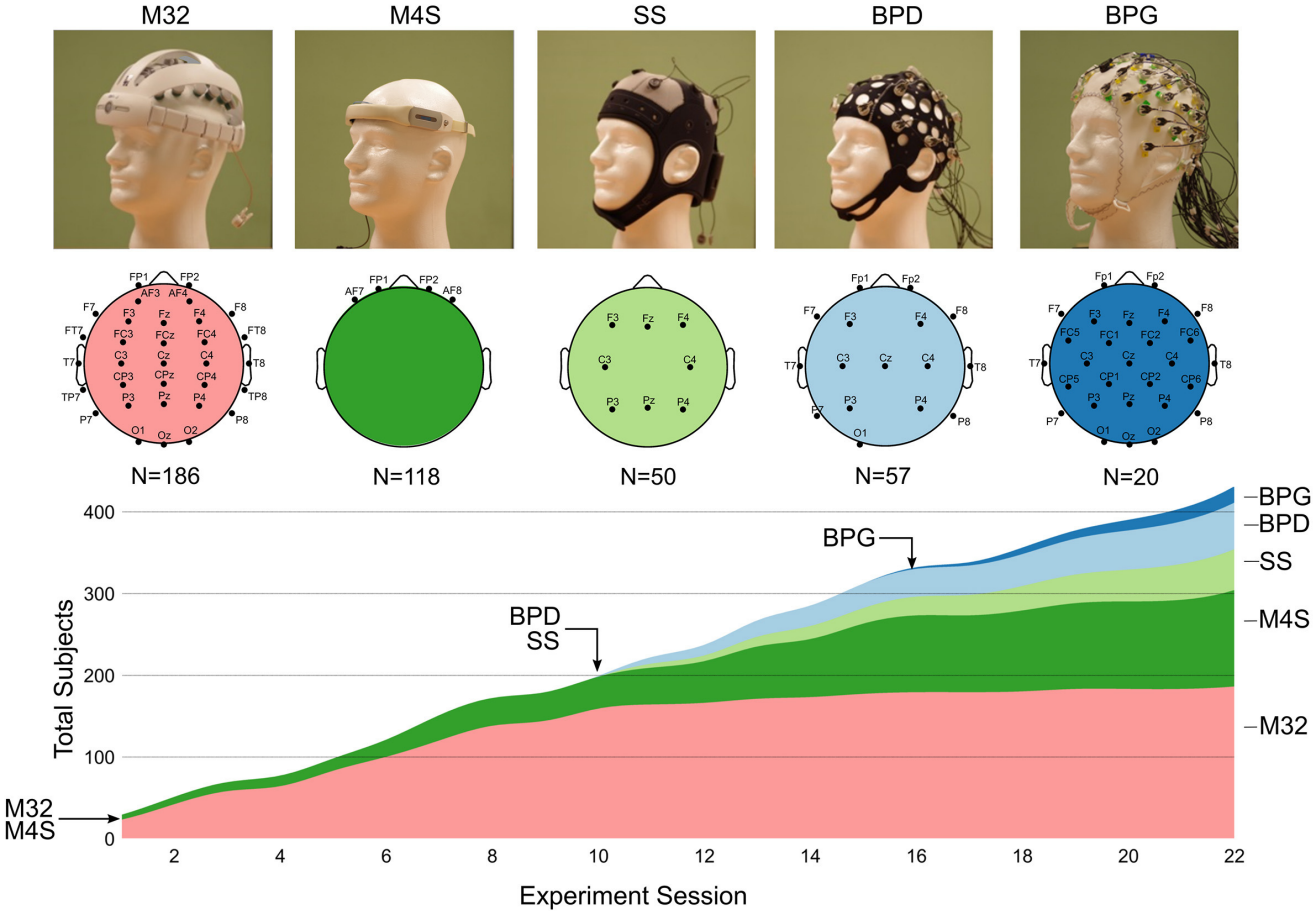
Image of each EEG system and their respective electrode montage. The chart shows the cumulative usage (stacked timeline) of each system over the course of 22 consecutive weekly experimental sessions. The arrows with headset names indicate the session number in which each device was introduced into the experiment. The total number of recordings with each headset is shown under the diagram of the electrode montage for each devices (*N* = number of subjects).

The volunteers were instrumented with one of four dry electrode EEG caps: Mindo-32 Trilobite (M32; National Chiao Tung University Brain Research Center^1^), Mindo-4S JellyFish (M4S; National Chiao Tung University Brain Research Center^2^), Neuroelectrics Starstim (SS; Neuroelectrics^3^), and Brain Products actiCAP Xpress (BPD; Brain Products GmbH)^4^. In addition, separate volunteers were recruited to participate in the experiment while wearing a traditional gel-based EEG cap: ActiCAP active electrodes with BrainAmp DC amplifier (BPG; Brain Products GmbH^5^). The dry electrode systems were loaned by the companies that manufactured them: two devices for M4S, four devices for M32, one device for SS, two devices for BPD, and one BPG system. The devices arrived at the experiment location at different times. Their cumulative usage is shown in Figure 1. Table 1 outlines the technical specifications of each of the systems.

**Table 1:** Technical specifications of the EEG systems used in this study.

**Parameter**	**Mindo-4S (M4S)**	**Neuroelectrics** **Starstim (SS)**	**actiCAP** **XpressV-Amp (BPD)**	**Mindo-32** **Trilobite (M32)**	**Brain Products** **BrainAmp DC (BPG)**
Number of subjects	12 (118)	33 (50)	28 (57)	43 (186)	18 (20)
Electrode type	Dry	Dry/Gel	Dry	Dry	Gel
Number of channels	4	8	16	32	32
Sampling rate (Fs) (Hz)	256	500	512	256	1,000
Ref/Gnd location	Mastoid	Neck/Mastoid	Earlobes	Earlobes	Earlobes
Bandwidth (Hz)	0.23–13k	DC−250	DC−500	0.23–13k	DC-1000
Input noise (μV_RMS_)	1.25	1.0	1.0	1.25	1.0
CMRR (dB)	110	115	100	110	110
I/P Impedance (MΩ)	3	1,000	100	3	10
Resolution (μV)	0.29	0.05	0.05	0.29	0.1
Input range (mV)	±2,400	±410	±410	±2,400	±3.28
Resolution (ADC)	24	24	24	24	16
Communication	Bluetooth	Bluetooth	Direct connection via USB to tablet/PC	Bluetooth	Direct to PC

*In the “number of subjects” entry, the total number of subjects that used this device is shown in parentheses, while the number of subjects retained for analysis is shown to the left of the parentheses*.

### Experimental tasks

After equipment set-up and prior to entering the exhibit, the participants were asked to sit facing a white wall with their eyes open for 1 min. Following the 1 min baseline, participants were encouraged to explore the exhibit for as long as they wished. Therefore, no constraints were placed upon the individual in relation to action, time, or navigation of the exhibit. The installation was contained in a 20 × 25 ft (6.1 × 7.6 m) room with two pieces in the center of the room and additional multi-media installations along the surrounding walls. The exhibit explored the history of the first recordings of the human heart beat and brain activity through a series of sculptures, installations, sound compositions, and a book. Following completion of the exhibit viewing, participants were asked to voluntarily complete a questionnaire summarizing their demographic information and outlining their preferred pieces in the installation regarding aesthetic appeal and emotional stimulation. Additional details, including more details of the exhibit and results of the questionnaire, can be found in (Kontson et al., 2015).

Permission was obtained from the artist to publish images of his work in this article. All other works are shown with permission from the Artists Rights Society and The Menil Collection.

#### Location tracking

One of three methods was used to track each individual's exploration of the art installation: manual annotation, annotation directly within the EEG files, and/or radio frequency identification (RFID). In the first method, the participants were manually tracked using a stopwatch that was synchronized with the beginning of the EEG recording. Their location and time of arrival was recorded each time a new art piece was being observed. In the second method, annotations were manually inserted into the EEG recording software to indicate the participant's location; this type of tracking was specific to the gel system. The third method utilized hand-held, wireless RFID readers that the participants used to scan RFID tags placed at various locations around the room. The time stamps associated with each location were recorded and saved for post-processing.

A location tracking method based on Bluetooth triangulation was explored as a fourth option for tracking. Unfortunately, due to poor spatial resolution, this method was unusable for data segmentation. Thus, only data with tracking information provided by the first three methods were used for analysis, which accounts for one half of the acquired data (192 subjects). A layout of the installation with examples of the art work at each location is shown in Supplementary Figure 2. Among these 192 subjects, the data from 58 subjects was excluded due to corrupted data files, resulting in a total of 134 subjects retained for analysis.

### Summary of EEG systems

The following sections provide a detailed description of the systems used in this study (shown in Figure 1. Image of each EEG system and their respective electrode montage. The chart shows the cumulative usage (stacked timeline) of each system over the course of 22 consecutive weekly experimental sessions. The arrows with headset names indicate the session number in which each device was introduced into the experiment. The total number of recordings with each headset is shown under the diagram of the electrode montage for each devices (*N* = number of subjects). All the dry electrode systems were loaned by the respective companies. The Brain Products ActiCAP with MOVE system was owned by the University of Houston. Table 1 provides an outline of the general technical specifications of each system.

#### Mindo trilobite (M32)

Mindo Trilobite is a 32-channel dry EEG system (Mindo, National Chiao Tung University Brain Research Center, Taiwan) that utilizes dry spring-loaded electrodes with foam-based sensors (Liao et al., 2011). EEG data are online referenced on the subject's earlobe. The headset is made of a plastic shell and has manual size adjustment mechanisms allowing for superior/inferior adjustment and circumferential adjustment around the head. The electrodes are organized according to the extended 10–20 international system. The individual electrodes are quickly attachable/detachable by a simple snapping mechanism. The device communicates wirelessly via Bluetooth to a tablet (MeMO Pad and Slate, ASUSTeK Computer Inc.) that is carried by the individual. Each tablet has a proprietary application installed for specific use with the Mindo devices. In these experiments, the data resolution was set to a maximum of 24 bits with a sampling rate of 256 Hz and saved as .cnt format. The data were automatically saved to the tablet and were recovered for analysis after the completion of each session.

#### Mindo 4S JellyFish (M4S)

The Mindo 4S JellyFish is a 4-channel dry EEG system (Mindo, National Chiao Tung University Brain Research Center, Taiwan). The reference and ground electrodes were placed on the side of neck using disposable adhesive electrodes. The headset is made of a plastic shell with a rubber band for adjustment around the head. The electrodes employ the same snapping mechanisms as in the Trilobite, allowing for rapid removal and replacement of individual electrodes. The 4S JellyFish electrode arrangement matches the four front-most electrodes of the 10-5 system (Oostenveld and Praamstra, 2001), the high resolution version of the international 10–20 nomenclature system, with an extra electrode on the outside for signal grounding. The system utilizes the same Bluetooth connection and graphical user interface as the Mindo Trilobite to transmit data to and store data on the portable tablet device. The same parameters were utilized for the 4S JellyFish as the Trilobite, with the exception of the number of channels: the data resolution was set to a maximum of 24 bits with a sampling rate of 256-Hz and saved as .cnt format.

#### Neuroelectrics starstim (SS)

Starstim is an 8-channel dry and gel, hybrid EEG and transcranial current stimulator (tCS) system from Neuroelectrics (Neuroelectrics, Barcelona, Spain). For the current study, only the recording feature of this system was used; no stimulation was applied to study participants. Although this system can be used both dry and with gel, the rigid Ag/AgCl dry electrodes were used exclusively in this study. The system is also fitted with a 3-axis accelerometer for recording of head acceleration. The scalp electrodes are labeled in accordance with the extended 10–20 international system. EEG data are online referenced directly on the subject's neck. The head cap is made of a flexible neoprene cap with a detachable battery and electrode-housing box. The device is wirelessly connected using Bluetooth 2.1 to tablets or PCs on which the software provided by the company is installed. For this experiment, the data were collected at a sampling rate of 256-Hz and wirelessly collected using a Microsoft Surface Pro (Microsoft Corporation, Richmond, WA).

#### Brain products actiCAP Xpress (BPD)

Brain Products actiCAP Xpress is a 16-channel dry EEG system (actiCAP system, Brain Products Xpress, Germany). The electrodes are worn in a flexible rubber cap and can easily be removed and replaced by fitting the electrodes within holes in the cap corresponding to the extended 10–20 international electrode nomenclature system. The dry sensors, known as Quick Bits, are flat T-shaped and round mushroom shaped electrodes for direct skin and scalp contact. The round shape of the mushroom-headed sensors allows for contact with the scalp without requiring the electrodes to be perpendicular to the head, while the T-shaped sensors allow direct skin contact with a high surface area for hairless and frontopolar positions. For these experiments, EEG data were online referenced on the subject's earlobe and the data were collected at a sampling rate of 512-Hz. The open-source 2015 version of the software OpenViBE (Renard et al., 2010) was used for development of an experiment-specific application. The EEG cap was wire-connected to a Microsoft Surface Pro (Microsoft Corporation, Richmond, WA) and the data were stored directly on the device during the experiment.

#### Brain products actiCAP with BrainAmp DC (BPG)

Brain Products actiCAP gel is an active gel-based EEG system (actiCap system, Brain Products GmbH, Germany) that can be used with 32, 64, or 128 channels. A total of 32 electrodes were utilized for this study. The electrodes were labeled in accordance with the extended 10–20 international system (Jurcak et al., 2007). EEG data were online referenced to channel FCz. In addition, two channels from the posterior peripheral channels (PO7 and PO8) were used to collect electrooculography (EOG) from below and on the temple of the right eye. All data were collected wirelessly at a sampling rate of 1,000-Hz. The BrainVision Analyzer software was used for all data collection, including manual tracking annotations.

### Data analysis

All data analysis was performed offline using MATLAB (The Mathworks Inc., Natick, MA). Raw EEG signals were manually segmented into windows of data from baseline and piece viewing (segmented by specific art piece) using the participant tracking methods outlined in the Location Tracking section.

#### Pre-processing and artifact removal

**Figure 2:**
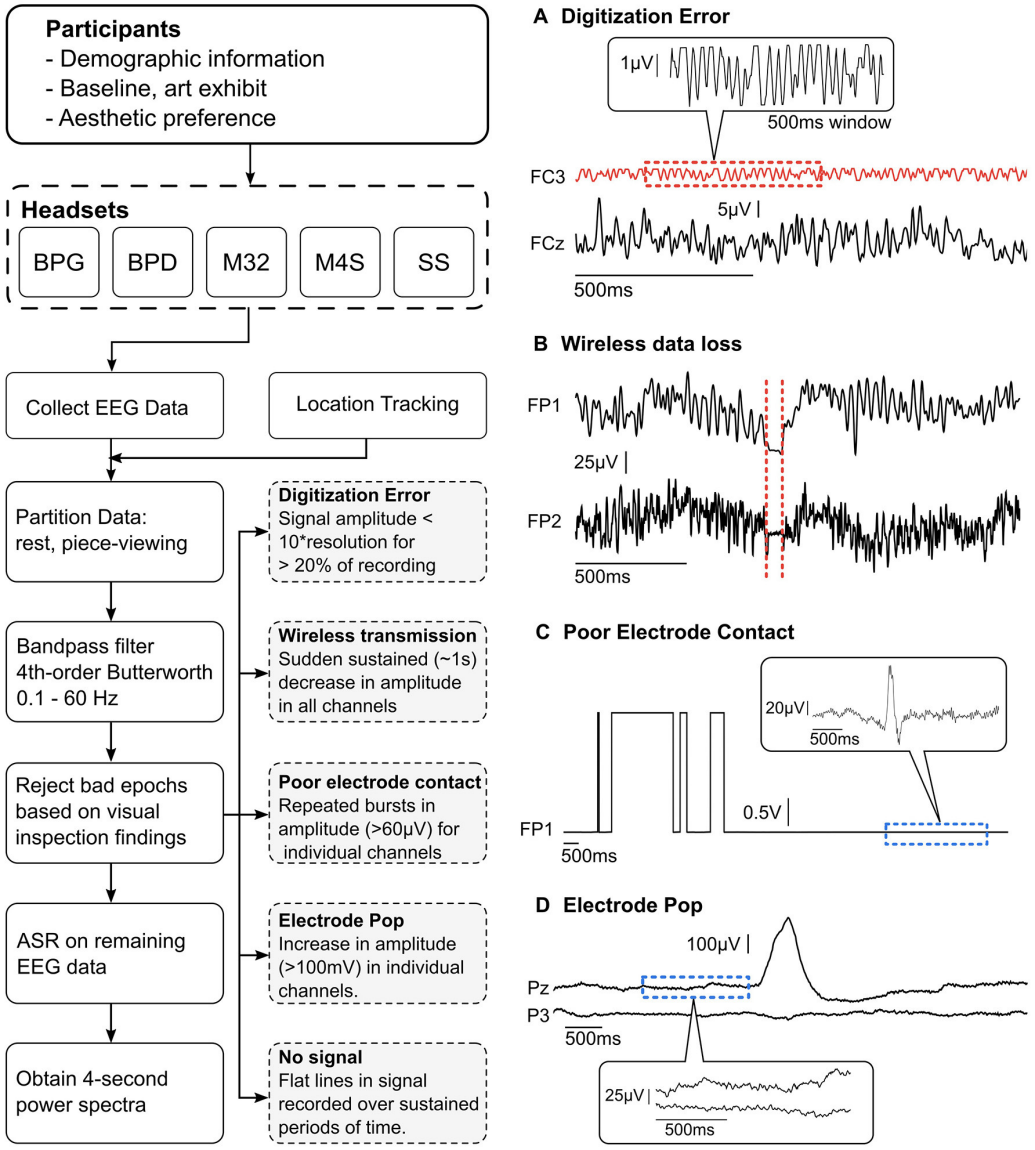
**(Left)** Data collection and pre-processing pipeline. Visual inspection was performed on all the partitioned data to find and reject bad epochs. The findings were used to create a set of guidelines (gray dashed boxes) to automatically remove bad epochs. **(Right, A–D**) Examples of the four most recurring gross artifacts encountered through visual inspection. The red dashed sections show artifactual segments of EEG data and the blue dashed sections show normal EEG data.

The data were high-pass filtered at 1 Hz to remove signal drift and low pass filtered at 50-Hz to avoid contamination from line-noise (60 Hz) using a 4th order zero-phase Butterworth filter. Initial visual inspection of the data revealed the presence of significant signal contamination from non-stereotypical, non-physiological artifacts. Although dry systems are becoming more present in the market and scientific literature, their overall signal characteristics, especially in non-laboratory settings, remains widely unexplored. Thus, for initial artifact characterization, it was necessary to manually identify and remove these segments of data. Five distinct, non-stereotypical and non-physiological artifact types were identified within the data sets: (1) poor electrode contact, (2) poor signal digitization due to low signal amplitude relative to amplifier resolution, (3) electrode pops, (4) data loss during wireless transmission, and (5) no signal (relative to the reference). Each of these artifact types is shown in Figure 2 and a detailed description of their characteristics is described in section Identification of non-physiological Artifacts.

Following the removal of the non-EEG artifacts, Artifact Subspace Reconstruction (ASR) (Mullen et al., 2013) was applied to further remove artifactual components from the data. ASR is an automated artifact rejection method available through EEGLAB (Delorme and Makeig, 2004). The algorithm uses a sliding window to identify segments of EEG data corrupted with artifacts. The algorithm first identifies regions of clean EEG data. Within each sliding window, principal components, which exceed a pre-defined threshold in terms of standard deviations, are identified as corrupted channels or segments of data. The corrupted data is reconstructed from neighboring channels using a mixing matrix, computed from the covariance matrix of the clean data, based on the volume conduction principle. For this study, as in the companion study (Kontson et al., 2015), a window length of 0.5 s and a threshold of three standard deviations were used. Given the potential for eye movement-induced increases in power of relevant EEG frequency bands, the ability of ASR to remove eye movement artifacts and other artifacts was previously compared to Independent Component Analysis (Kilicarslan et al., 2016). Results indicated that ASR performed similarly to ICA when identifying and correcting eye movements. The data were then re-referenced using the common average reference (CAR) (Delorme and Makeig, 2004; Garipelli et al., 2013).

#### Data segmentation

The denoised EEG data were partitioned manually into two conditions: (1) rest or baseline and (2) viewing of the artwork (piece viewing). The piece-viewing partition was based on the location tracking methods described in section Location Tracking. The staff's manual annotations with start and end time for viewing a particular piece by a subject within the museum exhibit, or the RFID activation times were used for the same purpose. The segmented data were further partitioned by sliding a window of 4 s with a 2 s overlap across each condition. Data segments with artifactual components, as described in section Identification of non-physiological Artifacts, were removed from further analysis.

#### Power spectral density (PSD)

The PSD for each data window was calculated using the Thompson's multi-taper PSD estimate: pmtm.m in Matlab (Mathworks, Natick MA) v. 2015b, where the tapers are the discrete prolate spheroidal sequences, time-halfbandwidth = 4, number of points = 512 (Thomson, 1982; Percival and Walden, 1993). The PSDs were obtained for each 4 s segment using 256 (downsampled) frequency bins between 1 and 50 Hz. The PSDs were normalized by dividing each 4 s PSD by its total power. The electrodes retained for analysis were based on the commonality across headsets. Electrodes Fp1 and Fp2 were common to BPG, BPD, M32, M4S. Electrodes F3, F4, C3, C4, P3, and P4 were common to BPG, BPD, M32, SS. Finally, electrode O1 was common to BPD, BPG, and M32. Overall, nine common electrodes were retained for further analysis based on the 10–20 standard for EEG channel locations: Fp1, Fp2, F3, F4, C3, C4, P3, P4, and O1. Figure 1 shows the electrode montage for each devices and Table 2 contains more information about the electrodes and number of EEG data segments analyzed for each headset.

**Table 2:** Number of samples *N*, after removing corrupted signals, corresponding to each electrode, and headset analyzed.

	**BPG**	**BPD**	**M32**	**M4S**	**SS**	**Total (*N*)**	**Kernel**	**σ**	***k* dominant eigenvalues**	***k* selected**
Fp1	3,085	845	1,327	460	—	5,717	Gauss	26	5	5
Fp2	3,085	845	1,349	460	—	5,739	Gauss	26	5	5
F3	3,085	832	1,349	—	1,415	6,681	Gauss	22	4	5
F4	3,085	841	1,349	—	1,404	6,679	Gauss	20	4	5
C3	3,085	845	1,349	—	1,415	6,694	Gauss	20	5	5
C4	3,085	845	1,343	—	1,380	6,653	Gauss	20	5	5
P3	3,085	845	1,349	—	1,415	6,694	Gauss	26	4	5
P4	3,085	845	1,349	—	1,372	6,651	Gauss	20	5	5
Total number of PSD segments	3,085	845	1,349	460	1,415					

*The value σ in the Gaussian Kernel corresponds to the best performing σ for each case. All clusters were performed by selecting k = 5 cluster centers to find*.

*“—” Indicates the headset did not contain that electrode. Gaussian kernel: $K\left(x,x^{\prime }\right)=\;exp\left(-\frac{||x-x^{\prime }|{|}^{\text{2}}}{\text{2}\sigma^{\text{2}}}\right)$, where ||*x*−*x*′||^2^ is the squared Euclidean distance between the two feature vectors, σ is a scaling parameter*.

#### Kernel K-means clustering

In this analysis, we sought not only to identify the usability of each system, but also the dominant spectral patterns within the EEG signals. We assessed the similarity of the frequency domain content of common electrodes using a data-driven unsupervised clustering approach. First, kernel K-means was implemented to find clusters of PSDs with similar characteristics across headsets and conditions (i.e., rest and piece viewing). The kernel function is a non-linear transformation that maps the input data into a higher dimensional Hilbert space to find better separability between data samples (Zhang and Rudnicky, 2002). The clustering was performed on 11 common electrodes separately: Fp1, Fp2, F3, F4, C3, C4, P3, P4, and O1. For each electrode analyzed, the 256 frequency bins were standardized by subtracting the mean and dividing by the standard deviation. The normalized spectra were used to construct the input matrix X_NxP_ where N is the data samples (see Table 2), and *P* = 256 standardized features.

Selecting the proper kernel function is critical in this clustering method. Several kernel functions were explored for this purpose: the linear kernel, polynomial kernel of degrees 2 and 3, and the Gaussian kernel with σ = [4, 12, 14, 20, 22, 24, 26, 28, 36, 44, 52, 60]. Furthermore, the number of selected clusters can influence the overall results. We employed a technique that allows reliable selection of the number of *k* clusters within the data by identifying the dominant eigenvectors of the non-linearly projected representation of the data, square symmetric Kernel matrix (Girolami, 2002). We computed the eigenvalue decomposition of the kernel matrix

$$\begin{array}{l} K_{NxN}=\;U\Lambda U^{T}, \end{array}$$

where the columns of *U* are the individual eigenvectors, *u*_*i*_, and Λ is a diagonal matrix containing the associated eigenvalues λ_*i*_. Now, we can write

$$\begin{array}{l} 1_{N}^{T}K1_{N}=1_{N}^{T}\left\{\overset{N}{\underset{i=1}{\sum }}\lambda_{i}u_{i}u_{i}^{T}\;\right\}=\;\overset{N}{\underset{i=1}{\sum }}\lambda_{i}{\left\{1_{N}^{T}u_{i}\right\}}^{2}. \end{array}$$

We identified the *k* number of clusters by selecting the *k* dominant terms in the summation $\lambda_{i}{\left\{1_{N}^{T}u_{i}\right\}}^{2}$ (Girolami, 2002). Electrodes Fp1, Fp2, C3, C4, and P4 showed *k* = 5 dominant terms in the eigenvalue decomposition, suggesting that the optimal number of clusters is ~5. Electrodes F3, F4, and P3 and O1 yielded *k* = 4 dominant terms. For consistency, *k* = 5 was chosen for the Kernel K-means algorithm to find five cluster centers. The overall highest performing kernel function (i.e., the function that produced the most well-distributed distribution of similarity values in the kernel matrix) was the Gaussian kernel with σ = (20, 22, 26) in all cases. The cluster assignment was initialized randomly 200 times, and the initialization configuration that produced the least within-cluster variance was selected as the best result in each case (Shawe-Taylor and Cristianini, 2004).

## Results

### Subject demographics

Supplementary Figure 1 shows the age and gender distribution of study participants as compared to the city of Houston's population distribution. The distribution of the participants follows the distribution of the Houston population with the exception of ages 6–10 years old. Ages below six were not eligible to participate due to poor fitting of headsets and inclusion/exclusion criteria approved by the IRB. The distribution of males and females who participated in the experiment was approximately evenly matched. Among the total number of participants, a total of 192 participants (95 male and 97 female) had annotated tracking data available for analysis. A location heatmap was created to visualize the patterns of movement within the installation space and the pieces that were viewed the most across all 192 subjects with tracking data (Supplementary Figure 2). The EEG data from 134 subjects was retained for further analysis due to data corruption in 58 subjects.

### Usability of each EEG system

The M32 headset was composed of a rigid plastic shell with simple manual adjustment mechanisms (small wheels that were adjusted by the fingers). This allowed for effortless placement on the subjects' head by simply donning it like a helmet, and adjusting the fit as necessary. However, one of the challenges faced with using the M32 system was the inability to maintain electrode contact due to poor fit on varying head sizes, and an inability to lock the adjustment mechanism. This resulted in gradual loosening of the fit over the period of usage and in many cases, constant electrode pops and amplifier saturation. The M32 headset was best suited for a rounder head shape; head shapes that were more ovular suffered from poor electrode contact on the lateral sides of the scalp (left: F7–P7; right: F8–P8) and on the left and right sides of longitudinal fissure (left: F3–P3; right: F4–P4). This is likely due to the fact that the adjustment mechanism only allowed for the increase and decrease of the radius of the cap and not for adjustment of the length and width independently. On the other hand, subjects with flat scalps suffered with poor contact on the superior surface only, likely occurring from a lack of adjustment on this axis. Differences in human head size and shape must be considered when designing an EEG system for the general population. As in Hairston et al. (2014), we found that stretchable EEG caps are desirable for optimal electrode placement and electrode contact. Custom 3D printed headsets may also help minimize poor fitting.

The M4S was composed of a hard plastic shell on which the electrodes were attached. Adjustment was accomplished using a flexible elastic band that could be loosened and tightened to accommodate the head size/shape of each subject, while still allowing for constant contact throughout the experiment. This system was certainly one of the most simple to adjust for subjects across ages, head shapes/sizes, and hair lengths. The electrodes were simply aligned to the anterior aspect of the scalp (forehead) and the elastic rubber strap was tightened into place.

The M32 and M4S systems suffered from a common issue that often resulted in poor signal quality and data loss: the electrodes were snapped into place using a standard 4-mm snap interface. Although this simplified the removal and replacement of individual electrodes, the snap could be easily undone when even small amounts of lateral pressure were applied to the electrode. In particular, when donning the system, users with long or thick hair could easily cause the electrodes to unsnap. In some cases, the subject might adjust the cap during the experiment, causing the electrodes to pop loose. This was less problematic with the M4S system; however, both were susceptible to this issue.

The 8-channel SS system employed a neoprene fabric cap that covers the entire head of the subject and is secured into place with a Velcro strap under the chin. The neoprene cap enabled simple and flexible donning/doffing and accommodated varying head sizes/shapes with a single cap. One of the drawbacks of this material is low permeability to air, resulting in excessive heat build-up under the cap and sweating by the subject. This can lead to electrodes shorting if sweat causes bridging. The spike style electrodes allowed for easy contact adjustment once the cap was secured into place. This resulted in better recording quality with low instances of electrode pop or poor contact. In some cases, subjects complained of discomfort with the spiky electrodes when used for extended periods of time. However, the number of instances and extent of discomfort were not documented and are thus not reported in further detail.

The BPD system utilizes a thin flexible rubber cap with rings for mounting metal dry electrodes for contact with the scalp. Due to the cap's flexibility, the placement and adjustment of the cap was simple and relatively quick. The electrodes themselves had long dome-shaped tips that helped to maintain contact when the electrode shifted to varying angles with respect to the scalp. The length of the tip could be easily adjusted by switching the tips for ones of the correct size. This feature helped to significantly improve comfort and initial fit; however, it was later observed that the dome shaped tips were highly susceptible to movement, resulting in significant system-related artifacts. The electrodes were individually wired to a splitter-box that was directly connected to a portable amplifier via a long ribbon cable. This allowed for a direction connection with the amplifier, however, it introduced the challenge of cable management behind the cap. The previous systems were all wired to a single point connected directly to the cap, reducing the need to consider pulling on the electrodes by the cables. The BPD system required conscious effort to ensure that electrodes were not free to move or be pulled, resulting in signal contamination by high amplitude artifacts. Nonetheless, when properly managed, the BPD system was simple to use and comfortable for the user over extended periods of time.

### Identification of non-physiological artifacts

After visual inspection of the data, it was determined that the presence of significant signal contamination from various artifacts warranted the manual identification, characterization, and removal of contaminated epochs from the data. Five distinct non-physiological artifact types were identified within the data sets: (1) electrode pops, (2) poor electrode contact, (3) digitization error resulting from low signal amplitude relative to the amplifier resolution, (4) data loss during wireless transmission, and (5) no signal (relative to the reference). Each of these artifact types is shown in Figure 2. These types of artifacts were identified using a thresholding technique based on manual identification of the artifacts in each system. The uniqueness of each system presented significant challenges when using an automated algorithm, and thus manual identification was selected as the preferred method for initial characterization of artifacts during data acquisition in natural complex settings.

After the artifacts were manually identified and characterized (Figure 2), we implemented an automatic gross artifact rejection scheme based on a threshold approach. An upper threshold of 300 μV was determined to be adequate for removal of high amplitude artifacts. In contrast to amplitude-based thresholding, higher order statistics (e.g., standard deviation, kurtosis) are commonly used in EEG experiments to select artifactual epochs of data to be removed, as proposed in Delorme et al. (2007). However, in our freely-moving environment with dry EEG electrodes, many of the sections of the data were highly contaminated with large-amplitude artifacts. These artifacts significantly distort the statistical properties of the data, making it challenging to obtain a true estimate of the higher order moments of the data. Thus, a threshold-based technique could be used across all systems since all reasonable EEG could be expected to occur below the given amplitude value. Specifically, an upper bound of 300 μV was used to remove high amplitude bursts. Brain-related EEG data has not been reported to reach 300 μV in amplitude. Typically, in experiments with little to no movement, the threshold amplitude is set at ~100 μV (Uriguen and Garcia-Zapirain, 2015). It is important to consider that dry EEG systems may require a higher amplitude threshold given the high impedance interface between the scalp and electrode; however, even in the case of dry EEG systems, 300 μV signals are likely to be artifactual regardless of the system used. This initial artifact identification method was employed to identify high amplitude bursts originating from non-physiological sources. As shown in Figure 2C, eye blinks can be clearly observed within the signal having amplitudes of ~100 μV, while the non-physiological bursts significantly exceed the 300 μV amplitude threshold. This is further illustrated in Figure 2D, where a single-channel electrode pop has an amplitude reaching ~400 μV, while the normal EEG signal is in the range of 1–100 μV.

A lower amplitude threshold was used to identify flat lines, digitization errors, and wireless data transmission errors. The lower threshold was set to 10 times the resolution of each system resulting in a system specific value for each headset: (1) M32: 3 μV, (2) M4S: 3 μV, (3) SS: 0.5 μV, and (4) BPD: 0.5 μV. The segment of data was removed if the amplitude exceeded the upper threshold, or the amplitude did not exceed the lower threshold in 20% of the recording. To ensure that contaminated epochs were fully removed from the data, 0.5 s before and after the labeled region were removed with the artifact. A final visual inspection was performed to remove epochs of wireless data transmission loss.

#### Poor electrode contact

Poor electrode contact presented as a similar artifact to electrode pops; however, the artifact was deemed to be poor contact if continuous segments of data suffered from repeated pops (shown in Figure 2C). It can be seen that the square shaped artifacts are on the order of volts. The magnified portion of the signal in the balloon shows actual signal containing an eye blink (~100 μV). Poor electrode contact was observed primarily in the M32 and BPD systems. In M32, poor contact was attributed to the rigid form factor, resulting in poor fit of the system. This was primarily due to head sizes exceeding the size of the rigid-body recording system, variable roundness, and local concaves in the head shapes. When a headset did not fit appropriately, the pressure may not be distributed over the scalp as intended, resulting in discomfort for the subject and displaced electrode locations (Hairston et al., 2014).

In BPD, poor electrode contact was observed when the dome shape electrodes would shift, causing the electrode to go in and out of contact as the subject moved around the space. In both systems, the artifact could be visually identified as large square waves that saturated at the peak, and repeatedly oscillated between low amplitudes and the maximum value. Of note, when the BPD system saturated, the output was orders of magnitude larger than the normal acceptable EEG signal.

#### Digitization error

The digitization error was an artifact that was observed to be specific to the M32 and M4S systems. This error presented as very low amplitude signals that had square-like waves. It was observed that the difference between values was near to the resolution of the analog-to-digital converter (ADC). Furthermore, the amplitude remained low throughout the entire trial. A close-up examination revealed that the signal is not in fact EEG (shown in Figures 2, **4A**).

#### Electrode pops

Electrode pops are historically well-documented artifacts that result from an instantaneous change in the scalp-interface potential, resulting in large amplitude (relative to the artifact-free EEG signal) bursts within the signal (Barlow, 1985). Although common in EEG, the presentation of these bursts, or pops, vary between headsets, depending on the electrode type, cause of the pop, and amplifier characteristics. Each of the headsets was examined for electrode pops and epochs exceeding the upper threshold were removed from the data. Electrode pops were identified as single events that were not connected to other such events directly (i.e., single bursts that start low, go high, and return to normal EEG amplitudes). This artifact was observed in all headset types.

#### Data loss during wireless transmission

Wireless transmission of data via Bluetooth or radio frequency can result in data loss if the connection between the transmitter and receiver is interrupted. In the case of this experiment, data loss during wireless transmission was observed when the subject was too far from the point of data reception, or if the transmission pathway was physically obstructed, either by the subjects' own body, or by other museum goers. This type of artifact was characterized by sudden and sustained reduction of amplitude across all channels. The last value was observed to be held until wireless transmission resumed. Figure 2B shows an example of this type of artifact, and how it was observed across all channels in the data. This type of artifact was observed rarely, but predominantly in the BPG wireless system.

#### Flat lines (i.e., no signal)

This type of artifact was simply described as flat lines, or no signal, in one or more channels; however, it was not considered an artifact if this occurred in all channels, but was instead identified as a failed recording. For this artifact type, the channel contained no data resulting from one of two primary reasons: (1) the electrode was not in contact with the scalp, or (2) the electrode had become disconnected and was not recording data. In the first case, improper connection resulted from a poor fitting cap (meaning the electrodes were not in contact with the scalp) or shifting of the cap during movement (causing the electrodes to lose signal during the experiment). In the second case, no signal would occur when the electrodes would become physically disconnected from the cap. This was unique to M32 and M4S, in which the electrodes would disconnect from the 4 mm snap connector due to the plug-in design of the electrodes, where the plug-in interface was facing the subjects' head.

**Figure 3:**
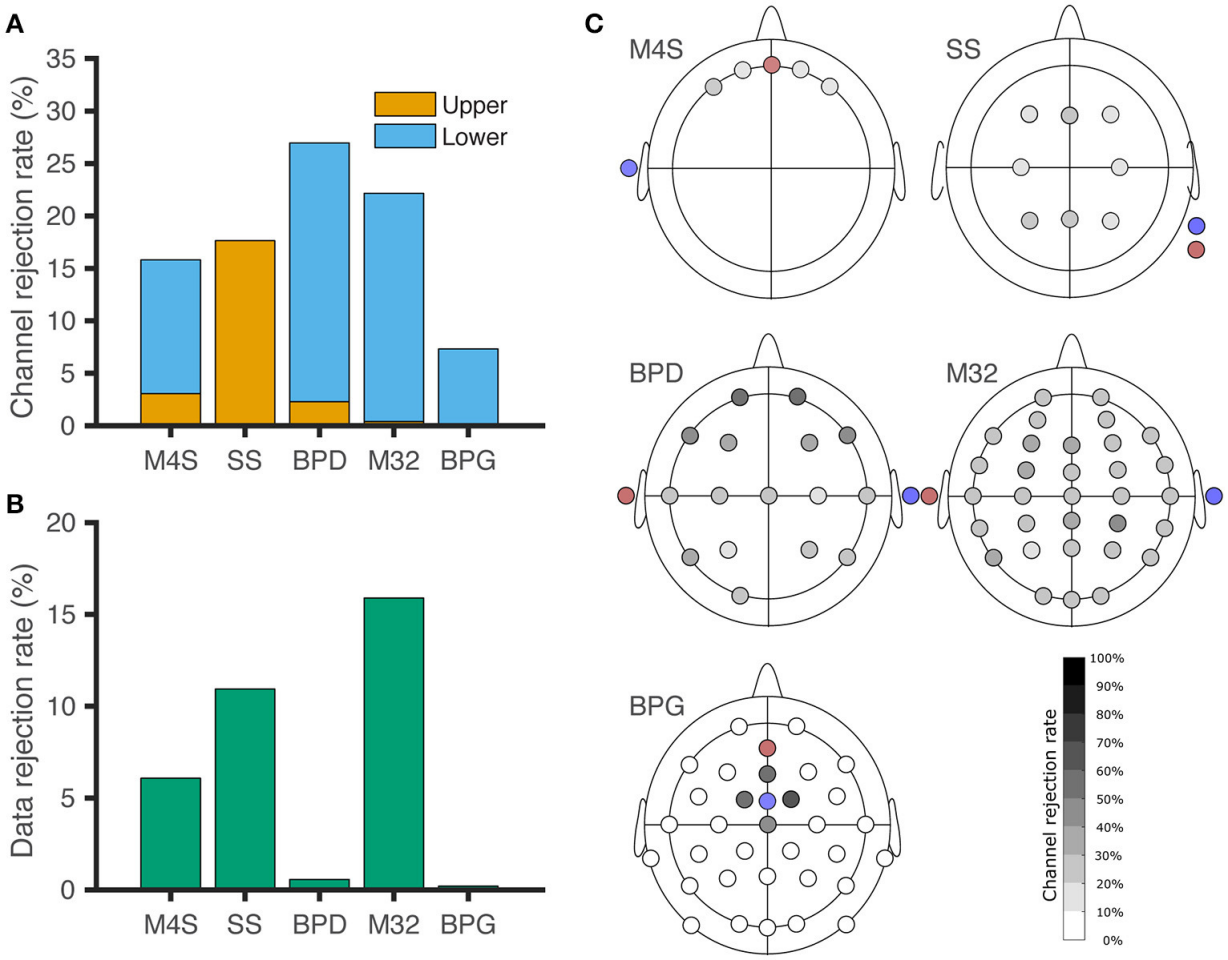
Channel and data rejection statistics for all the headsets. **(A)** Channel rejection rate across subjects during baseline and piece-viewing. The orange bars indicate the channel rejection rate due to the channel surpassing the upper threshold for 20% of the session, while the blue bars show the rejection rate based on the channel not surpassing the lower threshold for 80% of the session. **(B)** Data rejection rate across subjects during baseline and piece-viewing, after artefactual channel removal. **(C)** EEG channel rejection rate based on the amplitude thresholding criteria. Red: Ground. Blue: Reference. For BPG, the electrodes removed around the reference correspond to lower-threshold rejections, although those channels did not exhibit digitization errors.

Comparison among different headsets for channel and data rejection is represented in Figure 3. The channel rejection rate, from our criteria defined in section Identification of non-physiological Artifacts and Figure 2, was lowest for the -gel-based system BPG. For Figure 3A, windows of data where the recordings surpassed the upper threshold are associated with poor electrode contact (C) and electrode pops (D). Lower threshold rejection is associated with digitization error (A) for M4S and M32. For BPD and BPG, the amplitude resolution prevented digitization errors, but the channels were removed based on a one-fits-all scheme. In Figure 3B we analyze the data from the channels that were not removed based on the criteria. The windows of removed data in Figure 3B correspond to temporary poor electrode (C) contact and wireless transmission loss (B). BPD (0.57%) and BPG (0.2%) resulted in the least data rejection after removing artifactual channels.

### Clustering results: analysis of power spectra

The clustering analysis was performed as a method to evaluate the dominant spectral patterns within the EEG signals and to assess the similarity of the frequency domain content of common electrodes across the headsets. The kernel clustering analysis revealed five clusters of spectral patterns across all headsets for each of the electrodes in the sample analyzed. In general, the individual clusters showed typical spectral patterns, including PSDs with strong alpha (8–12 Hz) peaks, high beta (12–30 Hz) activations, and noise characteristics. We now describe gender, age, and piece-viewing specifics spectral patterns uncovered by the kernel clustering analysis.

**Figure 4:**
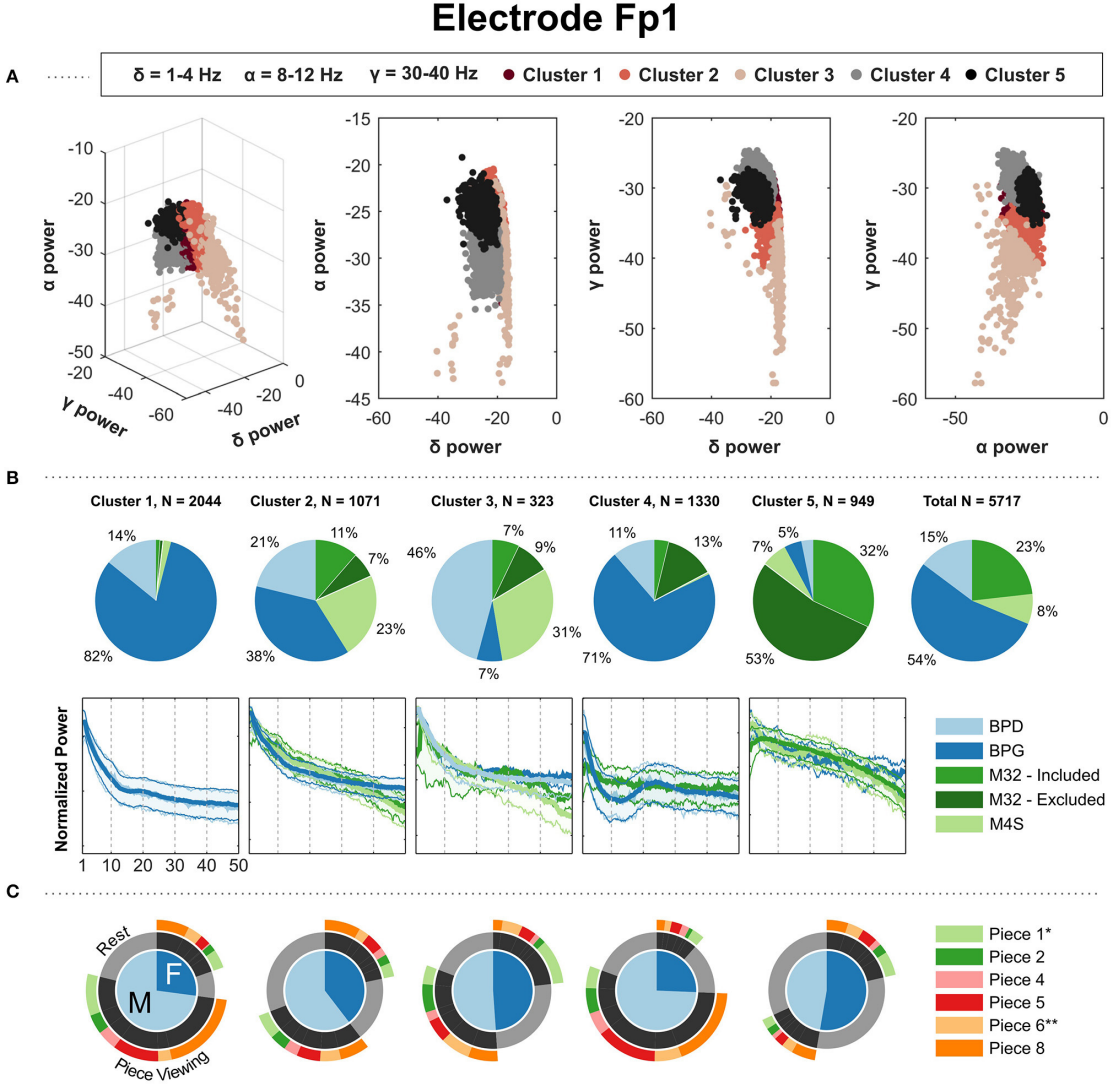
Results of kernel k-means clustering for electrode Fp1 (Gaussian kernel; σ = 26). **(A)** Three-dimensional visualization of the final clusters from kernel k-means. Each point in the scatter plot corresponds to the total normalized power (area under the PSD) in the delta, alpha, and gamma bands for a single 4 s window. **(B)** The pie charts show the contribution of each headset type to the PSD clusters. To the right, the last pie chart shows the overall distribution of the PSDs for each headset type. **(C)** The mean of the PSDs for each headset type is shown below each cluster's pie chart, along with the 5th and 95th percentiles as shaded regions. The PSDs from headset M32-A were excluded from visualization because they contain a prominent peak at 30 Hz from unknown source, not representative of the PSDs from headsets M32-B, M32-C, and M32-D. **(C)** Distribution of gender and condition information for the PDSs grouped in each cluster. ^*^Indicates most aesthetically pleasing and ^**^indicates most emotionally stimulating as reported in the questionnaire.

The spectral clustering for electrodes Fp1 (Figure 4) and Fp2 (Supplementary Materials) revealed a typical 1/f curve in the clusters that contain a majority of PSDs associated with headsets BPG, BPD, and M4S. The PSDs from M4S and BPD are grouped in the same cluster, with a 1/f shape in the curve. BPG was the dominant contributor to three clusters: Figure 4B, clusters 1, 2, and 4. Cluster 4 showed a high beta power increase, corresponding to a majority of the BPG-PSDs coming from “Piece-viewing” conditions. Cluster 1 shows a slight bump in the beta band also corresponding to a majority of BPG-PSDs coming from “Piece-viewing” conditions, and cluster 2 captures BPG-PSDs with a smooth 1/f curve with comparable number of samples coming from “Rest” and “Piece-viewing conditions.” There is a clear majority of M32 PSDs in cluster 5, indicating that the PSDs from the data taken using this particular headset was different than the other headsets: the PSDs in this cluster show a concave down characteristic curve.

**Figure 5:**
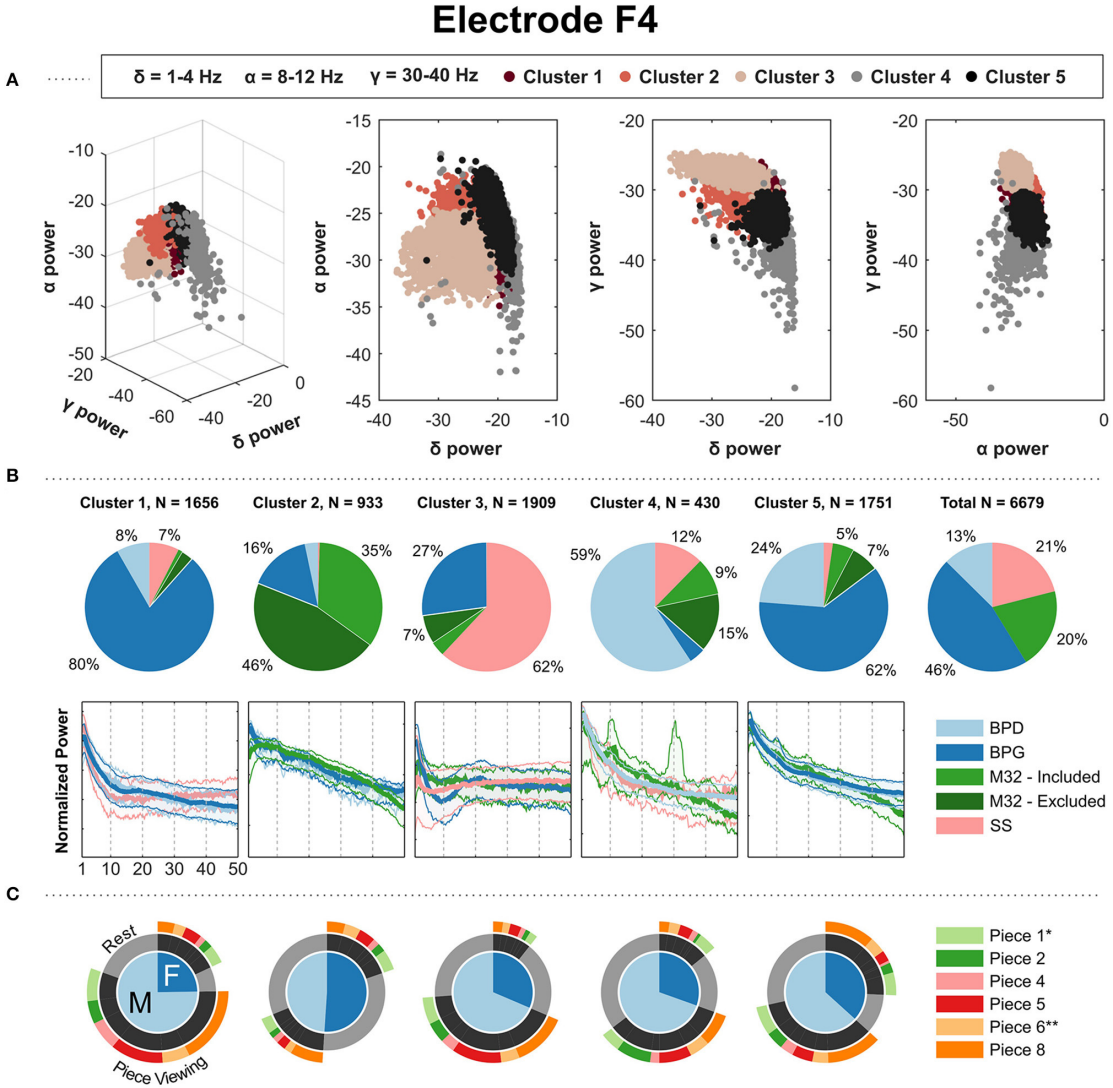
Gaussian kernel (σ = 22) k-means results for the parietal electrode F4. **(A)** Three-dimensional visualization of the final clusters from kernel k-means. Each point in the scatter plot corresponds to the total normalized power (area under the PSD) in the delta, alpha, and gamma bands for a single 4-s window. **(B)** The pie charts show the contribution of each headset type to the PSD clusters. To the right, the last pie chart shows the overall distribution of the PSDs for each headset type. **(C)** The mean of the PSDs for each headset type is shown below each cluster's pie chart, along with the 5th and 95th percentiles as shaded regions. The PSDs from headset M32-A were excluded from visualization because they contain a prominent peak at 30 Hz, not representative of the PSDs from headsets M32-B, M32-C, and M32-D. **(C)** Distribution of gender and condition information for the PDSs grouped in each cluster. ^*^Indicates most aesthetically pleasing and ^**^indicates most emotionally stimulating as reported in the questionnaire.

The clustering results from electrodes F3, F4, C3, C4, P3, and P4 showed similar results in terms of the composition of the clusters. Each cluster had a clear majority from one particular headset type, with two clusters corresponding to two major PSD shapes from BPG. Figure 5 shows the results for electrode F4. The results for electrodes F3, C3, C4, P3, and P4 are shown in the Supplementary Materials. In Figure 5, cluster 1 and 5 have a majority of BPG PSDs: both clusters show a 1/f curve, with cluster 1 containing a slight bump in the beta band. Cluster 2 contains a majority of the PSDs coming from M32, showing a concave down curve. Cluster 3 contains a clear majority of PSDs from SS, with the overall shape of them having a shape that rises and plateaus after ~5 Hz. Cluster 4 contains a majority of PSDs from BPD showing a 1/f curve, with similar-shape PSDs from SS and M32. The parietal electrodes, P3 and P4, yielded comparable results, with one of the BPG-majority clusters showing a more pronounced alpha peak corresponding with more PSDs from the “Rest” condition than the BPG-majority cluster with a smooth 1/f curve.

**Figure 6:**
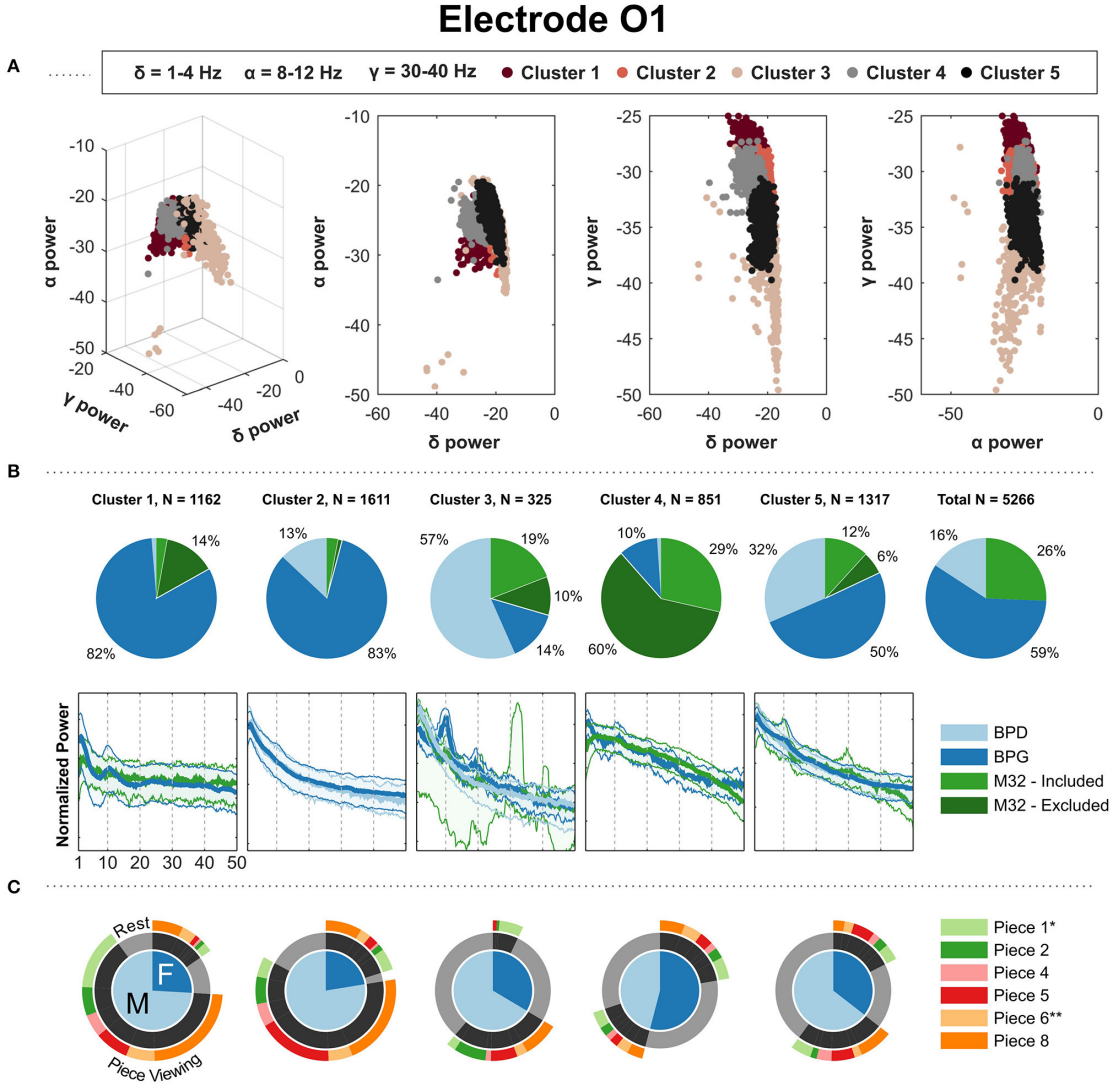
Gaussian kernel (σ = 20) k-means results for the parietal electrode O1. **(A)** Three-dimensional visualization of the final clusters from kernel k-means. Each point in the scatter plot corresponds to the total normalized power (area under the PSD) in the delta, alpha, and gamma bands for a single 4 s window. **(B)** The pie charts show the contribution of each headset type to the PSD clusters. To the right, the last pie chart shows the overall distribution of the PSDs for each headset type. **(C)** The mean of the PSDs for each headset type is shown below each cluster's pie chart, along with the 5th and 95th percentiles as shaded regions. The PSDs from headset M32-A were excluded from visualization because they contain a prominent peak at 30 Hz, not representative of the PSDs from headsets M32-B, M32-C and M32-D. **(C)** Distribution of gender and condition information for the PDSs grouped in each cluster. ^*^Indicates most aesthetically pleasing and ^**^indicates most emotionally stimulating as reported in the questionnaire.

The clustering on the occipital electrode O1 produced clusters pertaining to a headset-related majority. Figure 6 shows the clustering results for O1, as it was the only occipital electrode shared by at least three headsets. In Figure 6, clusters 1, 2, 5 showed a majority of PSDs taken from BPG: cluster 2 shows a smooth 1/f curve with the PSDs coming primarily from “Piece-viewing” conditions; cluster 1 shows a clear peak in the alpha band with PSDs coming mostly from “Piece-viewing” conditions; and cluster 5 shows a 1/f curve with bumps in the alpha band together with the headsets BPD and M32 with PSDs coming primarily from the “Rest” condition. BPD had a clear majority of PSDs in one cluster in which it's PSDs show a 1/f and come mainly from the “Rest” condition. However, the other contributing headsets, BPG (14%) and M32 (29%), show a strong peak in the alpha band. Finally, one cluster contains a majority of PSDs coming from M32, with a concave down shape.

## Discussion

In this study, we conducted a large-scale real-world experiment at the Menil Collection museum in Houston, Texas and acquired EEG data from 432 subjects using both dry and wet systems. Among these subjects, a total of 134 subjects had reliable tracking information and usable EEG data and were retained for analysis. We observed the distribution of the participants to be aligned with the population in Houston, where the experiments were conducted (Supplementary Figure 1). Trajectories of the participants were also visualized to identify the most viewed art pieces (Supplementary Figure 2). In addition, various types of distinct EEG artifacts that were commonly shown in the dry EEG headsets were identified, characterized, and described. Furthermore, kernel clustering was performed to characterize the EEG spectral patterns, to identify any differences among different EEG systems, and to assess differences in the features between baseline vs. piece-viewing and male vs. female.

In this freely-behaving approach, the EEG systems varied in their capacity to record characteristic modulations in the EEG data. The gel-based system clearly captured modulations in alpha and beta bands. We observed that high-beta power PSDs from pre-frontal and frontal electrodes came at a higher proportion from piece-viewing conditions than the baseline condition, and high-alpha power PSDs in central, parietal and occipital electrodes came at a higher proportion from the baseline condition. Although the patterns are also present in the dry-electrode systems (Figures 4B, 5B, 6B), the effects were suppressed and inconsistent (e.g., Figure 6B Cluster 3).

### Kernel K-means clusters

The clustering algorithms used on the normalized PSDs from the headsets analyzed resulted in five distinct clusters for electrode locations Fp1, Fp2, F3, F4, C3, C4, P3, P4, and O1. In most cases, the clusters contained a headset-specific majority, indicating that the overall shape of the PSDs depended on the system used, typically with two clusters pertaining to distinct PSD shapes from BPG. BPG was the only gel-based system used in this experiment, possibly accounting for the dominance of this headset in capturing data with consistent distinct PSD shapes. The four dry systems showed varying degrees of alpha and beta peak detection, which were clearly captured by BPG.

#### Data variability due to EEG systems used

These results indicate that the signal characteristics are dependent on the EEG system used. In addition, it was often the case that the dry electrode systems being clustered often with other dry electrode systems, excluding the gel electrode system. For this real-world EEG data collection, the recording system influenced the quality of the data collected. The systems used in this experiment varied in their capacity to record characteristic modulations in the EEG data, in particular for alpha and beta power. A recent study by Melnik et al. found that the subjects (32%) and the systems (9%) used for recording EEG data largely contributed to the total variance in the data (Melnik et al., 2017). This result comes from the comparison of three dry and one gel-based EEG systems on four subjects in short standard EEG tasks based on event-related potentials and steady-state visually evoked potentials.

In our experiment, the systems used were a significant source of variance in the data across hundreds of subjects in a freely-moving and richly stimulating environment. The high variance originating from the systems is likely due to significant variability in cap shape, electrode design, and amplifier characteristics. First, each system fits slightly different, causing certain channels to maintain better contact than others, while some may never reach the scalp (as in the rigid design of M32). Second, the electrode tips varied substantially in shape and material. The M32 electrodes used a combination of flat electrodes (frontal) and spring contact probes (M4S utilizes the same flat electrodes as the frontal channels in M32). The BPD system utilized a dome tipped electrodes to help maintain contact as the electrode rotates with respect to the scalp, while SS employs hard spiky electrodes to help penetrate through hair and maintain contact with the scalp. Lopez-Gordo et al. reviewed the various types of electrodes and observed significant differences between electrodes types in interface impedance, signal amplitude, and noise from external sources (e.g., motion, magnetic interference) (Lopez-Gordo et al., 2014). Thus, the signal quality assessment is highly influenced by these conditions. However, in our study, we do not seek to find the optimal configuration, but instead seek to assess the usability of these systems in an unconstrained setting.

#### Interpretation of PSD clusters

**Figure 7:**
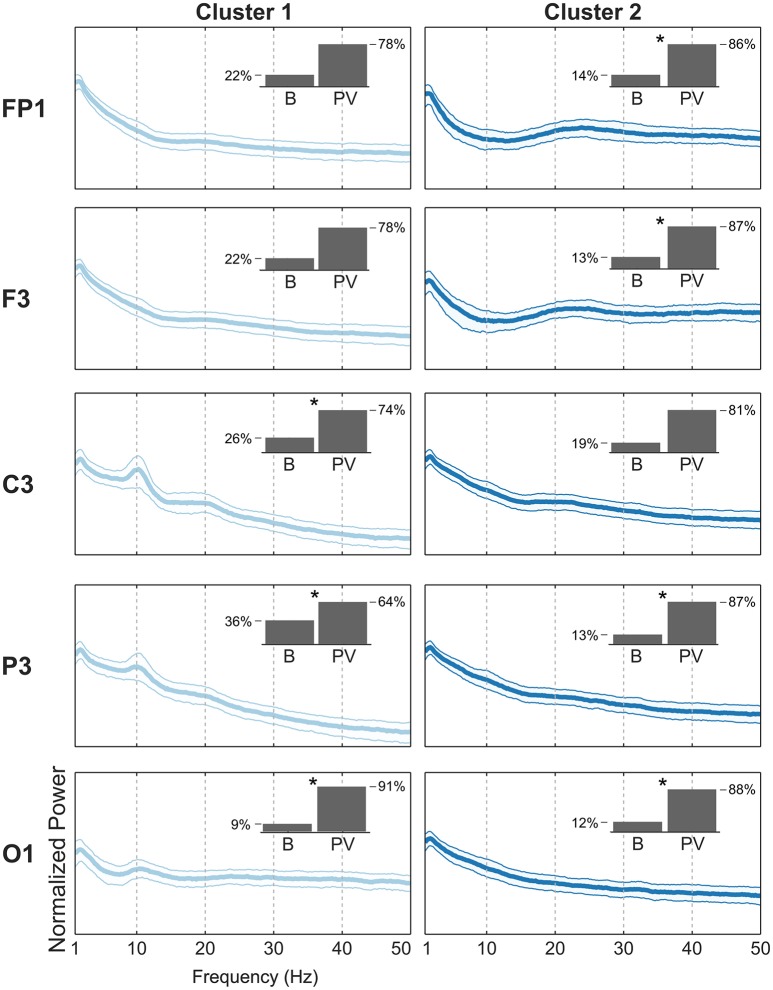
Comparison of PSDs from two BPG-majority clusters. Each row shows the median with the 20th and 80th percentiles of the PSDs from BPG grouped together in the BPG-largest clusters. **(Left)** PSDs from BPG in cluster 1. The inset shows the proportion of Baseline (B) and Piece-viewing (PV) PSDs in that cluster. An asterisk (^*^) indicates that there is a statistically significant difference with a confidence level of 99% between the cluster proportion of B vs. PV and the total sample proportion B (0.21) vs. PV (0.79) collected for BPG in the experiment. **(Right)** PSDs from BPG in cluster 2.

For the pre-frontal (Fp1, Fp2), frontal (F3, F4), and central (C3, C4) electrode locations, there were clear beta-band peaks with a preceding alpha-band valley in the BPG-PSDs grouped for the piece-viewing majority clusters (Figure 7). The beta peak was less prominent in the central electrode locations compared to pre-frontal and frontal. The beta band has been used in concentration tasks for experiments done outside the laboratory for massive EEG data collection (Umilta et al., 2012) as an indicator for concentration level of the subject or group of subjects. In the electrodes located over the parietal (P3, P4) and occipital (O1) regions, the BPG-PSDs with highest alpha-band power were grouped together in baseline-majority clusters (Figure 7). The high alpha peaks in the baseline condition, looking at a blank wall, may result from relaxation and lack of stimulating visual input in the subjects.

These observations of alpha suppression with beta peaks in PSDs coming from piece-viewing tasks in pre-frontal and frontal electrode locations, as well as alpha suppression in posterior electrodes, are consistent with the observations in the companion report (Kontson et al., 2015) in which high levels of connectivity were found between occipital and frontal scalp regions as the subjects experienced and evaluated the artistic stimuli in the room.

The contrary effects to our beta-band observations have also been reported in the literature. Umilta et al. reported no significant beta-band power changes as subjects observed abstract paintings on a computer screen (Umilta et al., 2012). The cognitive processes associated with the aesthetic and emotional experience of hundreds of subjects in natural settings (e.g., museums, galleries, artist studios) is naturally expected to be different. For each individual, there will be a multitude of processes (or internal states) and brain regions involved in the aesthetic experience (Dietrich and Kanso, 2010) and understanding of the artwork exposed. The analysis exposed here does not attempt to inquire into the particularities of each subjects' aesthetic experience. Rather, we grouped the data into natural clusters that revealed overall PSD properties and how they relate to “baseline” vs. “piece-viewing” tasks, gender, or recording system used. Instead, we point the reader to Kontson et al. (2015), where a subset of EEG data (specifically from BPG) was analyzed in detail.

### Usability and future implications

This section provides a qualitative analysis of the usability of each system as perceived by the researchers.

#### Donning/doffing and fit of EEG cap

A previous study testing the usability of different commercially available EEG systems in which subjects moved freely in a rich-stimulation environment (Hairston et al., 2014) found that the form factor and manufacturing materials provide different levels of comfort and electrode location due to different head shapes and forms. We found that it is difficult to accommodate varying head shapes and sizes using EEG systems with hard shells. For extended periods of time, this could be ameliorated with custom 3D printed hard-shells that fit the user's head anatomy while maintaining the headset in place.

#### Application interface, data recording, and portability

Each of the systems varied in how the data were recorded, including wireless transmission or direct connection to a PC or tablet. This feature played a direct role in the reliability of the recordings and the degree of portability that each system possessed. The M32 and M4S systems interfaced directly with an android application that came pre-loaded on a small tablet that was provided by the company (MeMO Pad, ASUSTeK Computer Inc.). The devices were synchronized with the tablet via Bluetooth and all recording settings were established directly through the application interface. Depending on the subject's preferences, the tablet was either carried throughout the duration of the experiment, or placed on a small table near the entrance of the room. The wireless connection allowed the system to be portable, requiring the user to occupy only one hand with the tablet or to be completely hands-free if chosen (in the case of the tablet remaining on the table). One drawback of this system was unreliability of the user interface: the tablet application was observed to crash unexpectedly, stopping data recording for the remainder of the subject's session. However, the interface itself was easy to use and provided the user immediate visual representation of the signal.

The SS system was a highly portable system that communicated directly from a module on the cap to a wireless USB dongle that was plugged into a tablet (with USB connections) or PC. In this experiment, the Microsoft Surface Pro 3 tablet (Microsoft, Inc.) was used to allow for increased portability. The data were transmitted wirelessly and recorded on the Neuroelectrics Instrument Controller (NIC) software. NIC was observed to be very stable and did not result in data loss due to crashing or software failure. Similar to the M32 and M4S systems, all recording settings, including initiation and termination of the trial, were established through the software.

The BPD system was the least portable of the systems requiring a direct connection between the amplifier and host recording system (either PC or tablet with USB connection). To maximize portability, the amplifier of the BPD system was connected to a Microsoft Surface Pro 3 tablet (Microsoft, Inc.) and the data were recorded on a custom application using the open-source BCI software, OpenViBE (Renard et al., 2010). It should be noted that this system is also capable of interfacing directly with the manufacturer's data collection software, BrainVision Analyzer. As a result of the cabling between the electrodes/amplifier and amplifier/tablet, the subjects were asked to wear a small backpack in which they stored the tablet for the duration of the experiment. One issue encountered with both the BPD and SS systems was the limited amount of time that the Surface Pro could be used for collecting data. Constant use over extended periods of time resulted in rapid battery depletion and excessive heat build-up, often causing the tablets to shut down in the middle of data. However, this was a challenge associated with the recording device (Surface Pro) and does not directly reflect the performance of the EEG systems. Thus, similar studies conduct in the future should account for these problems.

## Limitations of the study

The EEG systems were obtained on loan from the respective companies, thus they were used as they arrived to the experiment location (Figure 1). As a result, there was an uneven number of datasets for each of the headsets. The real-world experimental setting, in which museum-goers as volunteers, resulted in the laboratory staff procuring limited set-up time to prepare the subject with the EEG system. An additional limitation of this study was the variability in the EEG headsets, including shape, electrode material/shape, data transmission method, and amplifier properties. However, in this study, we sought to identify patterns within the data that are expected to exist in all EEG signals (e.g., 1/f power spectrum, changes in alpha rhythms, etc.). Additionally, we did not seek to identify an optimal configuration of any of these features, but rather report on the overall performance of these commercial systems.

Preliminary analyses did not show a clear segmentation of the clustered PSDs based on age, gender, occupation, race/ethnicity or gender. We decided to illustrate the distribution of gender within each cluster (Figures 4C, 5C, 6C), but age, occupation, race/ethnicity were not covered in this report. Additionally, the results of the questionnaire given at the end of the exhibit visit (e.g., most aesthetically pleasing piece, most emotionally stimulating piece) were not addressed in this report.

## Author contributions

JB, JGC-G, SN, KK, and MM contributed in collecting data at Menil Collection. JB, JGC-G, and SN contributed equally analyzing the data, interpreting the results, and writing the manuscript. DR contributed the use of his artwork and facilitating the study at the Menil Collection. JLC-V conceived and directed the research, and edited the manuscript.

### Conflict of interest statement

The authors declare that the research was conducted in the absence of any commercial or financial relationships that could be construed as a potential conflict of interest. The EEG systems were loaned at no cost to the University of Houston for the duration of the experiment. The respective companies were contacted to ensure the correct operation of the headsets. Brain Vision LLC (Morrisville, NC) recently joined as an in-kind member of the IUCRC BRAIN University of Houston Site (JLC-V).

## Footnotes

^1^M32; National Chiao Tung University Brain Research Center, T. Mindo-32 Trilobite [Online]. Available online at: http://mindo.com.tw/en/goods.php?act=view&no=17 (Accessed June 30th 2017).

^2^M4S; National Chiao Tung University Brain Research Center, T. Mindo-4S Jellyfish [Online]. Available online at: http://mindo.com.tw/en/goods.php?act=view&no=4 (Accessed June 30th 2017).

^3^SS; Neuroelectrics, B., Spain Starstim 8 [Online]. Available online at: http://www.neuroelectrics.com/products/starstim/starstim-8/ (Accessed June 30th 2017).

^4^BPD; Brain Products GmbH, G., *Germany Brain Products actiCAP Xpress* [Online]. Available online at: http://www.brainproducts.com/actiCAPXpress (Accessed June 30th 2017).

^5^BPG; Brain Products GmbH, G., *Germany Brain Products BrainAmp DC* [Online]. Available online at: http://www.brainproducts.com/productdetails.php?id=2 (Accessed June 30th 2017).
