# Supplementary material for: Deployment of Mobile EEG Technology in an Art Museum Setting: Evaluation of Signal Quality and Usability
Source: Front Hum Neurosci. 2017 Nov 10;11:527. doi: 10.3389/fnhum.2017.00527 (PMC5686057; doi:10.3389/fnhum.2017.00527)
Supplement: Supplementary file 1 [file DataSheet1.docx]

Supplementary Material

**Deployment of Mobile EEG Technology in an Art Museum Setting: Evaluation of Signal Quality and Usability**

**Jesus G. Cruz-Garza^1,*,†^, Justin A. Brantley^1,†^, Sho Nakagome^1,†^, Kimberly Kontson^2^, Murad Megjhani^3^, Dario Robleto^4,5^, Jose L. Contreras-Vidal^1^**

^1^Laboratory for Non-Invasive Brain Machine Interfaces, Department of Electrical and Computer Engineering, University of Houston, Houston, TX, USA

^2^ Division of Biomedical Physics, Office of Science and Engineering Laboratories, Center for Devices and Radiological Health, U.S. Food and Drug Administration, Silver Spring, MD, USA

^3^Department of Neurology, Columbia University, New York, NY, USA

^4^Artist in Residence in Neuroaesthetics, Cullen College of Engineering, Houston, TX, USA

**^†^**These authors contributed equally to this work

*** Correspondence:**
Corresponding Author: Jesus G. Cruz-Garza
[jgcruz@uh.edu](mailto:jgcruz@uh.edu)

# Subject demographics – population distribution and tracking results

**Supplementary Figure 1** shows the age and gender distribution of study participants as compared to the city of Houston’s population distribution. We see that the distribution of the participants follows the distribution of the Houston population with the exception of ages six to ten years old. Ages below six were not eligible to participate due to poor fitting of headsets and inclusion/exclusion criteria approved by the IRB.

The distribution of males and females who participated in the experiment was approximately evenly matched. Among the total number of participants, a total of 192 participants (95 male and 97 female) had annotated tracking data available for analysis. A location heatmap was created to visualize their trajectory within the installation space (**Supplementary Figure 2 A**). In this figure, the color intensity provides an indication of the percentage of people who visited each of the pieces, while the dotted gray line displays their trajectory within the space. The art pieces with the highest percentage of visitors were localized around the entrance, likely due to the fact that all participants had to pass through that area. The most viewed pieces were six (97%), eight (96%), one (94%), three (91%), and five (90%), while the least visited piece was four, with only 57% of participants visiting this piece. The artwork pieces are depicted in **Supplementary Figure 2 A**. The figure also shows results from questionnaires about “Emotionally Stimulating Art Pieces” and “Aesthetically Pleasing Art Pieces” which are shown as histograms in **Supplementary Figure 2 B) and C)**. The most emotionally stimulating piece was the piece index 6 and the most aesthetically pleasing piece was the piece index 1 which corresponds to the numbering in the same figure A).


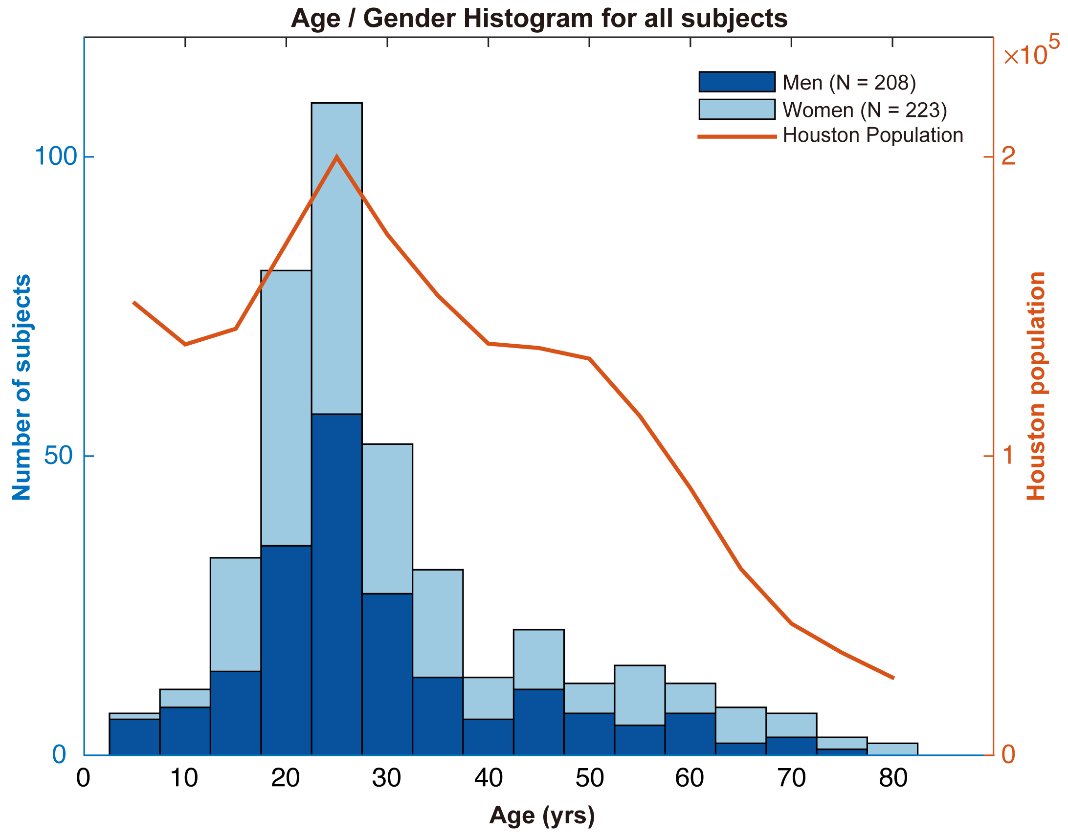


Supplementary Figure 1. Distribution of study participants separated by gender (Male: dark blue; Female: light blue). The subject pool is shown relative to the population of the greater Houston area.


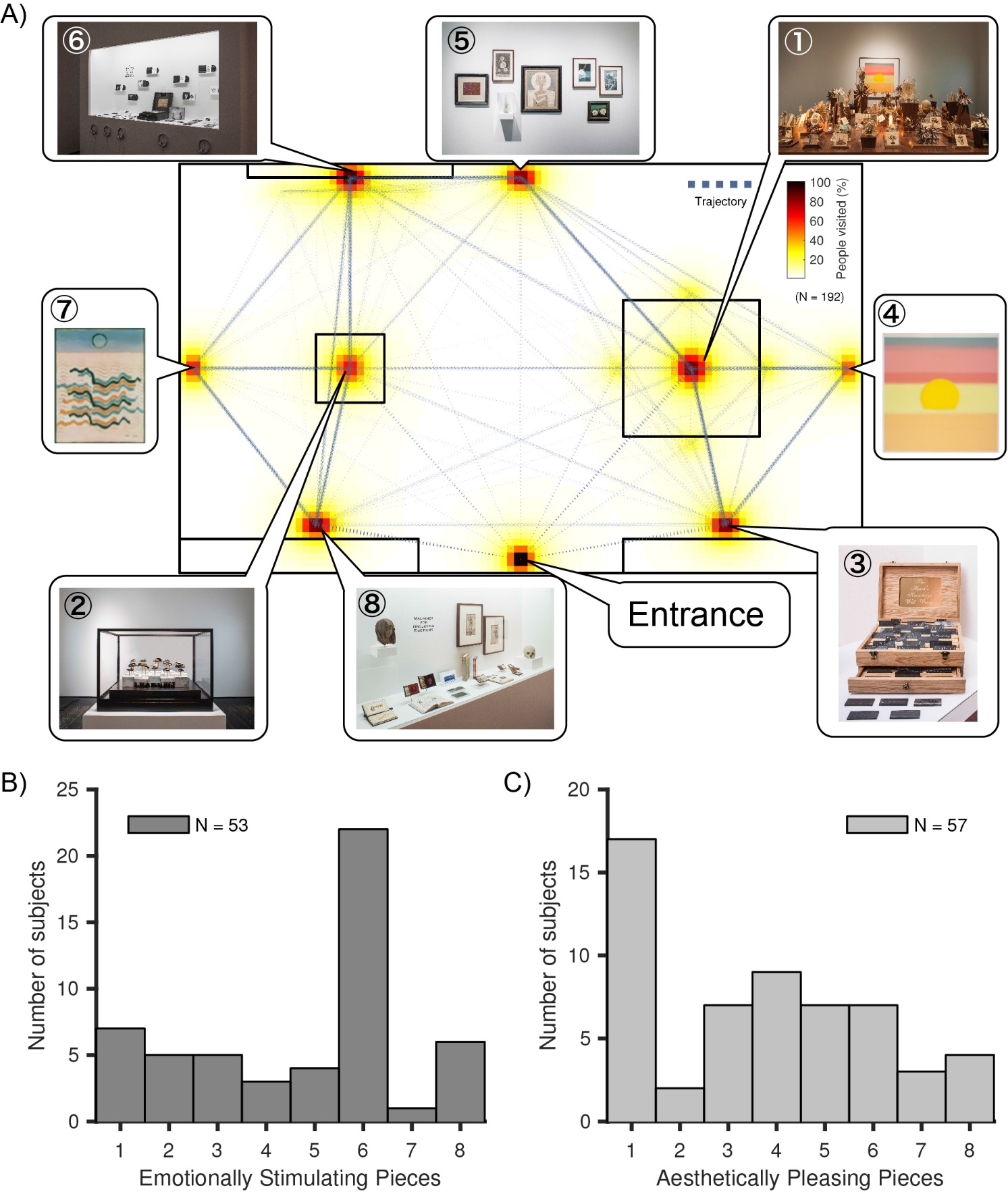


**Supplementary Figure 2.** Location heat map showing the distribution of participants at each of the pieces within the exhibit space (detailed view of the artwork is shown in the bubbles) with histograms generated from questionnaire answered by some of the subjects. A) The total number of participants represented in this figure is N = 192 (Male = 95, Female = 97). The gray dotted line indicates trajectories from one piece to another. B) A histogram of emotionally stimulating pieces answered by 53 subjects within 192 subjects considered in A). The number on the x-axis corresponds with the piece number indicated in the above heat map figure A). C) A histogram of aesthetically pleasing pieces answered by 57 subjects within 192 subjects considered in B). The number on the x-axis corresponds with the piece number indicated in the figure A).

# PSD clustering analysis

The kernel k-means clustering was performed for electrodes Fp1, Fp2, F3, F4, C3, C4, P3, P4, and O1 in the international 10-20 system EEG channel locations. The results of the clustering for electrodes Fp1, F4, and O1 are shown in the main article body. The following figures show the results of clustering in all the other channels represented in the original manuscript.


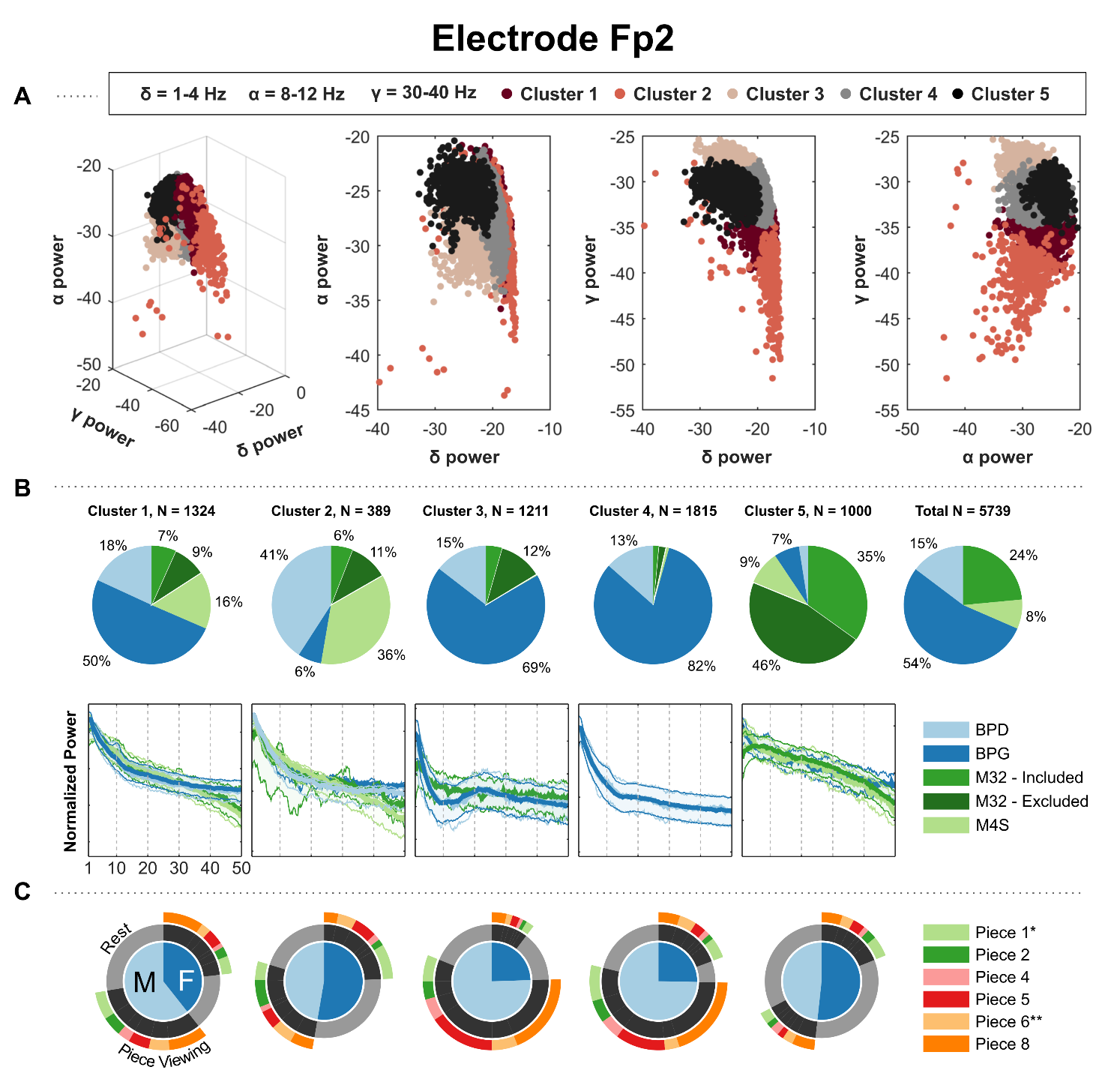


**Supplementary Figure 3.** Results of kernel k-means clustering for electrode Fp2 (Gaussian kernel; σ = 26). A) Three-dimensional visualization of the final clusters from kernel k-means. Each point in the scatter plot corresponds to the total normalized power (area under the PSD) in the delta, alpha, and gamma bands for a single 4-second window. B) The pie charts show the contribution of each headset type to the PSD clusters. To the right, the last pie chart shows the overall distribution of the PSDs for each headset type. C) The mean of the PSDs for each headset type is shown below each cluster's pie chart, along with the 5th and 95th percentiles as shaded regions. The PSDs from headset M32-A were excluded from visualization because they contain a prominent peak at 30Hz from unknown source, not representative of the PSDs from headsets M32-B, M32-C and M32-D. C) Distribution of gender and condition information for the PDSs grouped in each cluster. *Indicates most aesthetically pleasing and **indicates most emotionally stimulating as reported in the questionnaire.

**
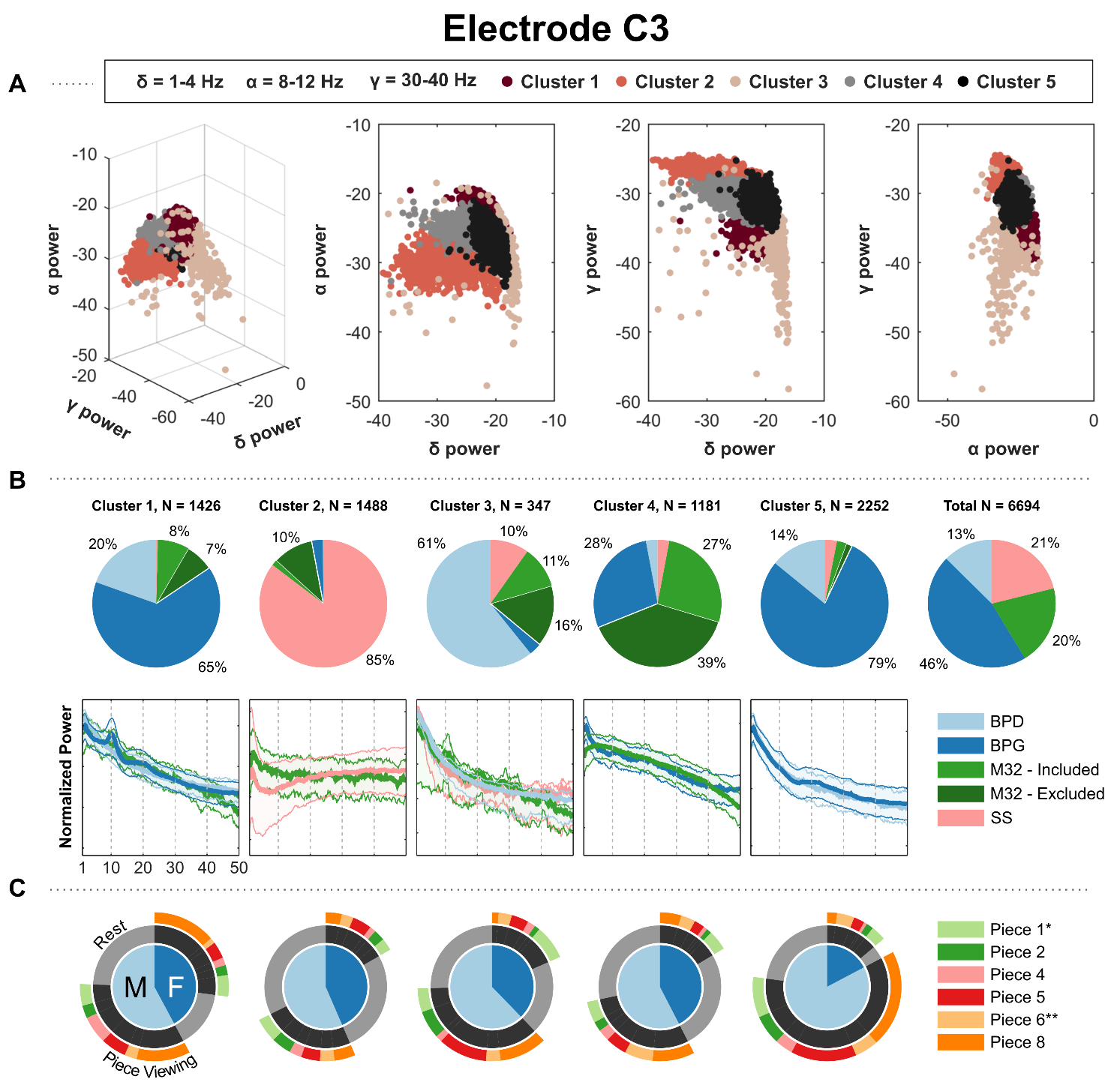
**

**Supplementary Figure 4.** Results of kernel k-means clustering for electrode C3 (Gaussian kernel; σ = 26). A) Three-dimensional visualization of the final clusters from kernel k-means. Each point in the scatter plot corresponds to the total normalized power (area under the PSD) in the delta, alpha, and gamma bands for a single 4-second window. B) The pie charts show the contribution of each headset type to the PSD clusters. To the right, the last pie chart shows the overall distribution of the PSDs for each headset type. C) The mean of the PSDs for each headset type is shown below each cluster's pie chart, along with the 5th and 95th percentiles as shaded regions. The PSDs from headset M32-A were excluded from visualization because they contain a prominent peak at 30Hz from unknown source, not representative of the PSDs from headsets M32-B, M32-C and M32-D. C) Distribution of gender and condition information for the PDSs grouped in each cluster. *Indicates most aesthetically pleasing and **indicates most emotionally stimulating as reported in the questionnaire.


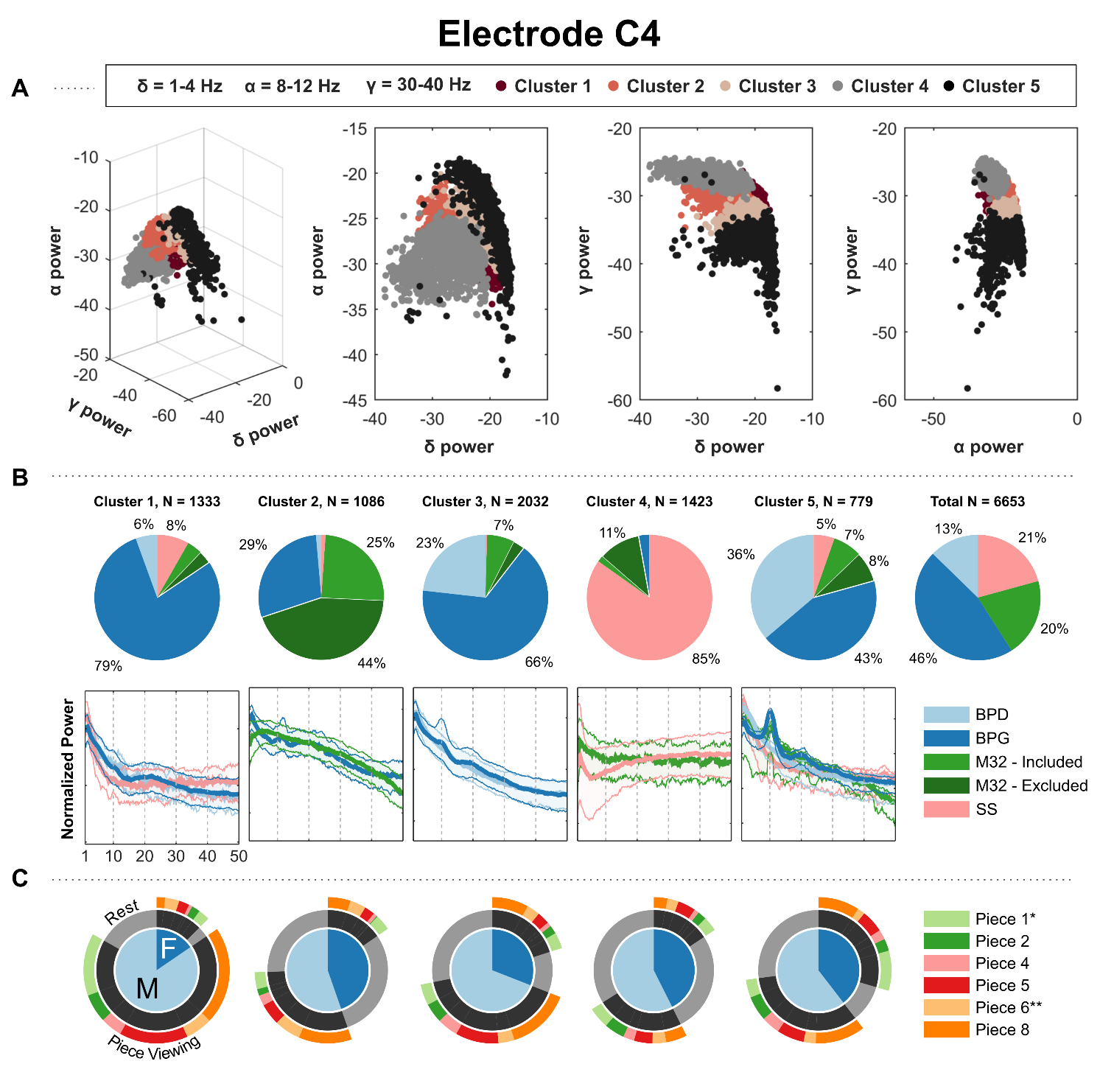


**Supplementary Figure 5.** Results of kernel k-means clustering for electrode C4 (Gaussian kernel; σ = 26). A) Three-dimensional visualization of the final clusters from kernel k-means. Each point in the scatter plot corresponds to the total normalized power (area under the PSD) in the delta, alpha, and gamma bands for a single 4-second window. B) The pie charts show the contribution of each headset type to the PSD clusters. To the right, the last pie chart shows the overall distribution of the PSDs for each headset type. C) The mean of the PSDs for each headset type is shown below each cluster's pie chart, along with the 5th and 95th percentiles as shaded regions. The PSDs from headset M32-A were excluded from visualization because they contain a prominent peak at 30Hz from unknown source, not representative of the PSDs from headsets M32-B, M32-C and M32-D. C) Distribution of gender and condition information for the PDSs grouped in each cluster. *Indicates most aesthetically pleasing and **indicates most emotionally stimulating as reported in the questionnaire.


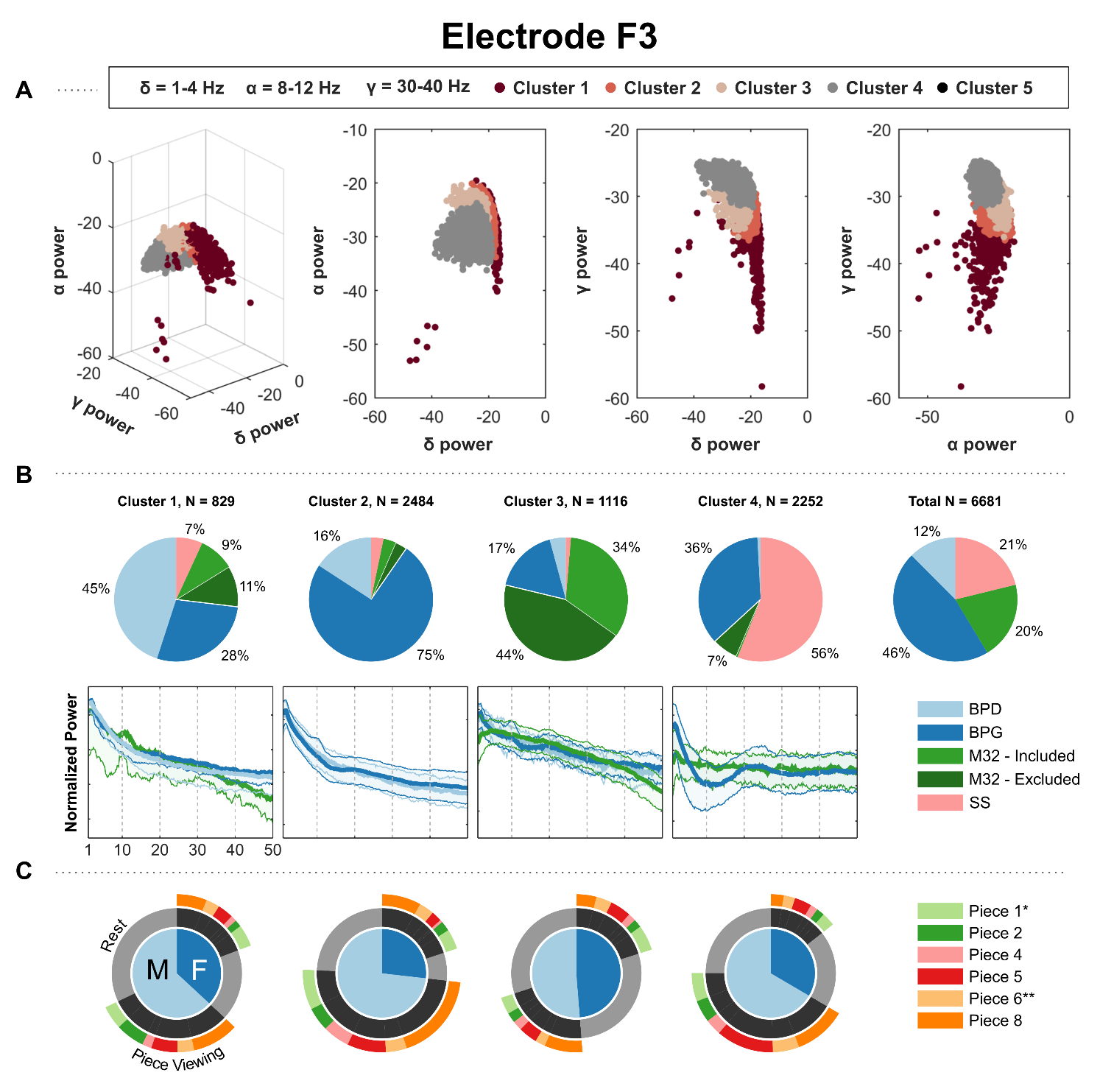


**Supplementary Figure 6.** Results of kernel k-means clustering for electrode F3 (Gaussian kernel; σ = 26). A) Three-dimensional visualization of the final clusters from kernel k-means. Each point in the scatter plot corresponds to the total normalized power (area under the PSD) in the delta, alpha, and gamma bands for a single 4-second window. B) The pie charts show the contribution of each headset type to the PSD clusters. To the right, the last pie chart shows the overall distribution of the PSDs for each headset type. C) The mean of the PSDs for each headset type is shown below each cluster's pie chart, along with the 5th and 95th percentiles as shaded regions. The PSDs from headset M32-A were excluded from visualization because they contain a prominent peak at 30Hz from unknown source, not representative of the PSDs from headsets M32-B, M32-C and M32-D. C) Distribution of gender and condition information for the PDSs grouped in each cluster. *Indicates most aesthetically pleasing and **indicates most emotionally stimulating as reported in the questionnaire.


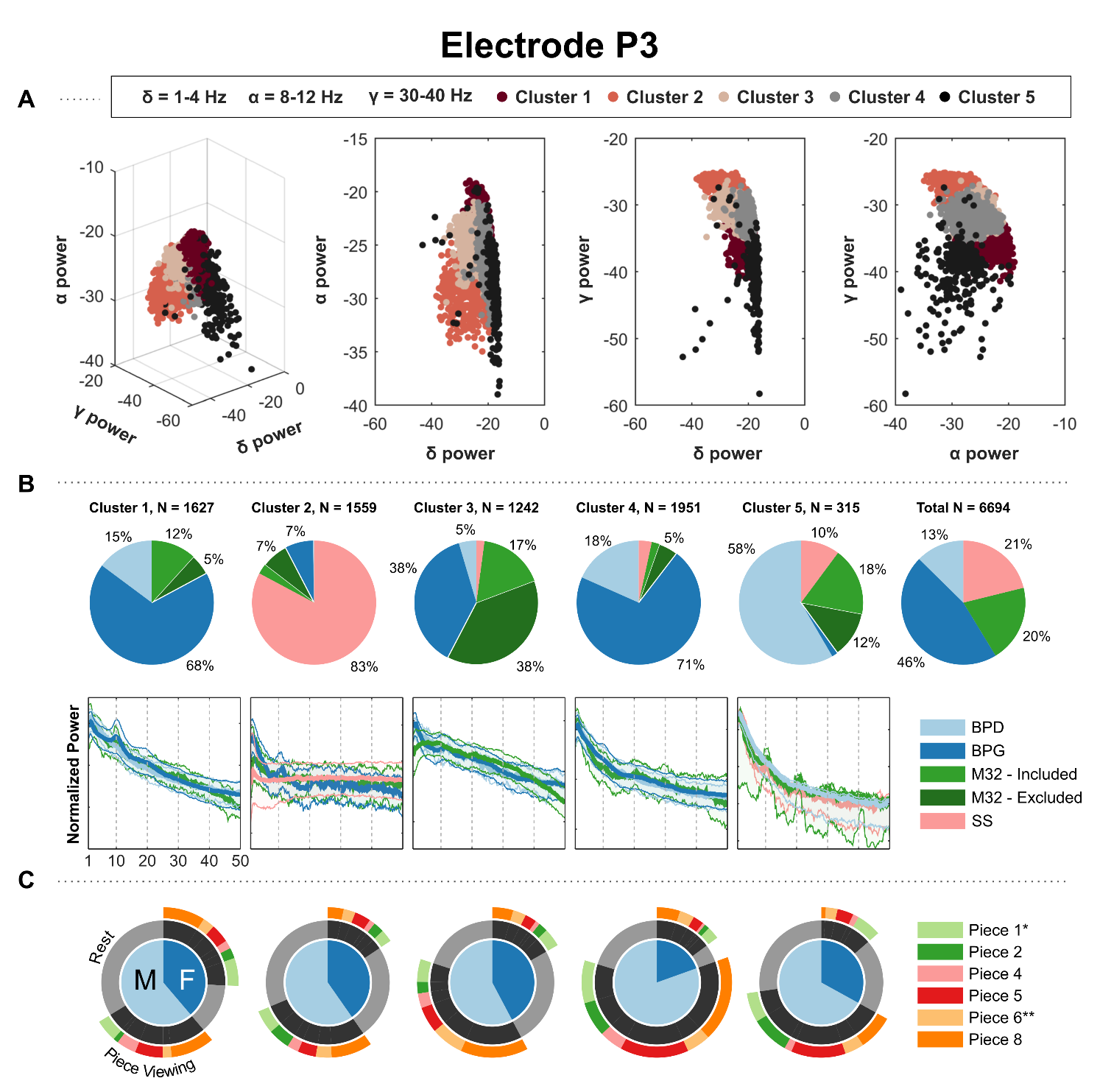


**Supplementary Figure 7.** Results of kernel k-means clustering for electrode P3 (Gaussian kernel; σ = 26). A) Three-dimensional visualization of the final clusters from kernel k-means. Each point in the scatter plot corresponds to the total normalized power (area under the PSD) in the delta, alpha, and gamma bands for a single 4-second window. B) The pie charts show the contribution of each headset type to the PSD clusters. To the right, the last pie chart shows the overall distribution of the PSDs for each headset type. C) The mean of the PSDs for each headset type is shown below each cluster's pie chart, along with the 5th and 95th percentiles as shaded regions. The PSDs from headset M32-A were excluded from visualization because they contain a prominent peak at 30Hz from unknown source, not representative of the PSDs from headsets M32-B, M32-C and M32-D. C) Distribution of gender and condition information for the PDSs grouped in each cluster. *Indicates most aesthetically pleasing and **indicates most emotionally stimulating as reported in the questionnaire.


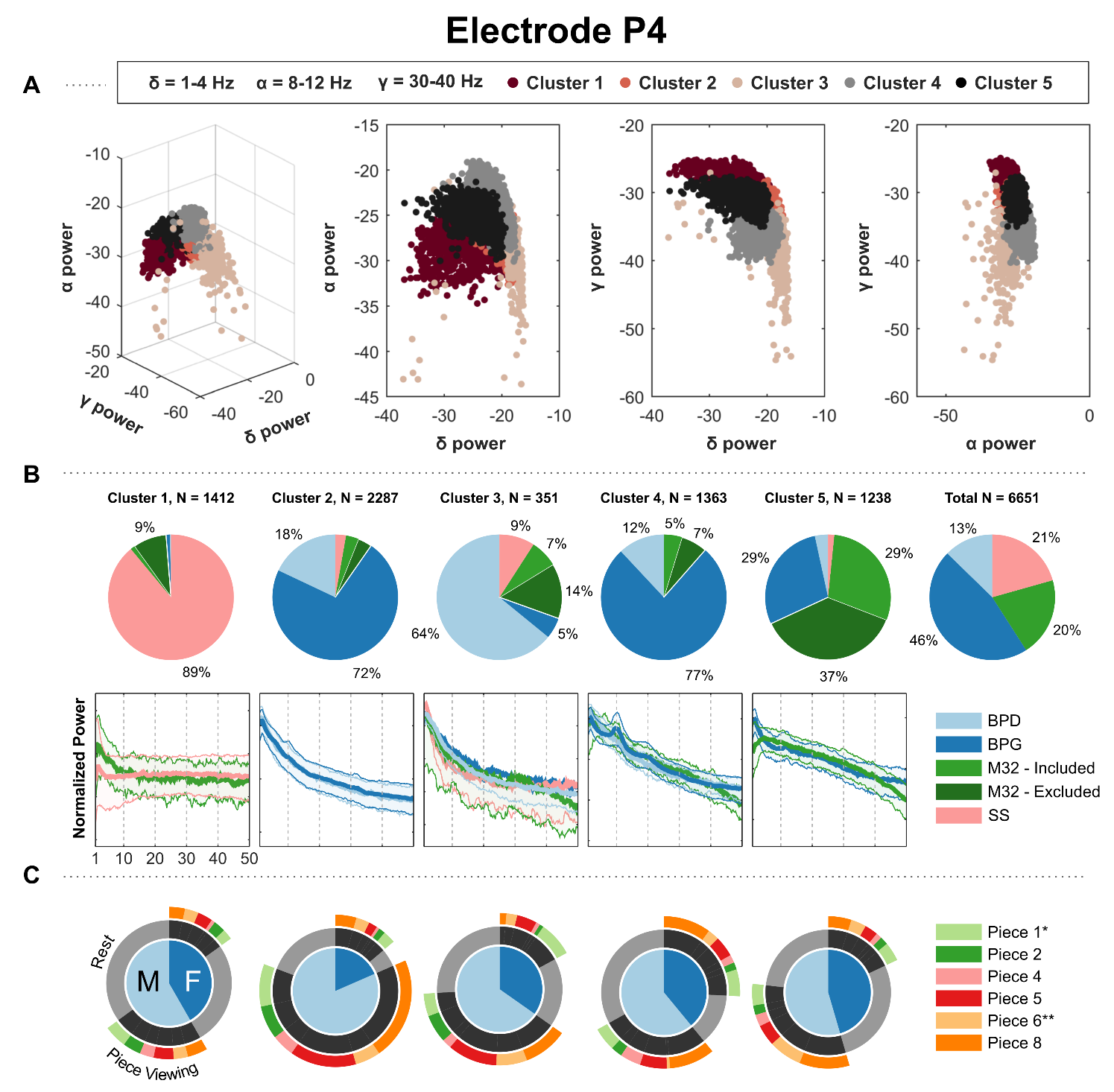


**Supplementary Figure 8.** Results of kernel k-means clustering for electrode P4 (Gaussian kernel; σ = 26). A) Three-dimensional visualization of the final clusters from kernel k-means. Each point in the scatter plot corresponds to the total normalized power (area under the PSD) in the delta, alpha, and gamma bands for a single 4-second window. B) The pie charts show the contribution of each headset type to the PSD clusters. To the right, the last pie chart shows the overall distribution of the PSDs for each headset type. C) The mean of the PSDs for each headset type is shown below each cluster's pie chart, along with the 5th and 95th percentiles as shaded regions. The PSDs from headset M32-A were excluded from visualization because they contain a prominent peak at 30Hz from unknown source, not representative of the PSDs from headsets M32-B, M32-C and M32-D. C) Distribution of gender and condition information for the PDSs grouped in each cluster. *Indicates most aesthetically pleasing and **indicates most emotionally stimulating as reported in the questionnaire.
